# Research Progress of Plant-Derived Natural Products against Drug-Resistant Cancer

**DOI:** 10.3390/nu16060797

**Published:** 2024-03-11

**Authors:** Wenli Liu, Yuqin Wang, Lijie Xia, Jinyao Li

**Affiliations:** Xinjiang Key Laboratory of Biological Resources and Genetic Engineering, College of Life Science and Technology, Xinjiang University, Urumqi 830046, China; 107552100970@stu.xju.edu.cn (W.L.); wangyuqin@stu.xju.edu.cn (Y.W.)

**Keywords:** plant-derived natural products, cancer, drug resistance, molecular mechanisms

## Abstract

As one of the malignant diseases globally, cancer seriously endangers human physical and mental health because of its high morbidity and mortality. Conventional cancer treatment strategies, such as surgical resection and chemoradiotherapy, are effective at the early stage of cancer but have limited efficacy for advanced cancer. Along with cancer progress and treatment, resistance develops gradually within the population of tumor cells. As a consequence, drug resistance become the major cause that leads to disease progression and poor clinical prognosis in some patients. The mechanisms of cancer drug resistance are quite complex and involve various molecular and cellular mechanisms. Therefore, exploring the mechanisms and finding specific targets are becoming imperative to overcome drug resistance. In recent years, plant-derived natural products have been evaluated as potential therapeutic candidates against cancer with drug resistance due to low side effects and high anticancer efficacy. A growing number of studies have shown that natural products can achieve superior antitumor effects through multiple signaling pathways. The mechanisms include regulation of multiple drug resistance (MDR)-related genes, inhibition of the phosphatidylinositol 3-kinase (PI3K)/protein kinase B (AKT) signaling pathway, induction of autophagy, and blockade of the cell cycle. This paper reviews the molecular and cellular mechanisms of cancer drug resistance, as well as the therapeutic effects and mechanisms of plant-derived natural products against cancer drug resistance. It provides references for developing therapeutic medication for drug-resistant cancer treatment with high efficacy and low side effects.

## 1. Introduction

Cancer is one of the most dominant causes of death in the world. It is estimated that more than 19.3 million new cases have been diagnosed with 10 million deaths annually worldwide [1]. The gender gap for the incidence rate of all cancers, being 19% higher in men than in women in 2020, although there are wide differences in the distribution across regions [2]. Moreover, the burden of cancer incidence and mortality is rapidly growing around the world. Currently, the main cancer treatment strategies include surgical resection [3], radiotherapy, chemotherapy [4], immunotherapy [5], and targeted therapy [6]. Although the conventional therapies are effective at the early stage of cancer, they have limited efficacy for locally occurring and metastatic cancer due to severe side effects, drug resistance, multiple recurrences, and metastases [7,8,9].

A major reason for treatment failure in cancer patients is the resistance to chemotherapeutic agents [10]. Drug resistance to therapies in cancer can be classified as primary or acquired. Primary resistance refers to the ability of cancers to escape initial therapy. And acquired drug resistance develops after continuous exposure to a chemotherapeutic drug, even though the drug is initially active [11]. When the body develops resistance, the response to these drugs will decline. The body gains a cross-resistance to a variety of chemotherapeutic drugs with different cellular targets and functions, which is called multiple drug resistance (MDR) [12]. It is ineffective and toxic to use a large doses of drugs to overcome multidrug resistance [13]. Studies have shown that multiple factors, such as epigenetics, micro RNAs (miRNAs), and long-stranded noncoding RNAs (lncRNAs), contribute to the development of multidrug resistance (MDR) in cancer cells [14,15,16,17]. Therefore, it is increasingly important to understand the molecular mechanisms that lead to the development of drug resistance, which has now been elucidated for many cancers and allows the use of conventional chemotherapeutic anticancer agents to cause DNA damage to kill drug-sensitive cancer cells [18,19]. However, in order to overcome drug resistance in cancer cells, drugs that can be delivered to specific target molecules must be developed to improve therapeutic accuracy. Therefore, there is an urgent need to develop therapeutic agents with a better safety profile and higher efficacy for drug-resistant cancer treatment.

For the past few years, plant-derived natural products have been evaluated as the most potential candidates for drug-resistant oncology therapies. They can overcome drug resistance with low side effects. A variety of plant-derived natural products with anti-drug-resistant-tumor activities have been identified, such as alkaloids, terpenoids, phenols, flavonoids, which can inhibit expression of resistant protein [20], suppress tumor-cell invasion and migration [21], induce apoptosis [22], and restrain angiogenesis and proliferation [23]. Natural products can work better when combined with other anticancer drugs. Studies have demonstrated that flavonoids in combination with suitable anticancer drugs have improved their therapeutic indexes by increasing their bioavailability, thereby reducing lethal side effects by lowering the dose of chemotherapeutic agents [24]. It was proven that MDR in gastric cancer reversed by curcumin is closely related to NF-κB-mediated apoptosis [25,26], and the Chinese herb glaucine, an isoquinoline alkaloid isolated from the stem of Corydalis yanhusuo, can inhibit P-gp and MRP1 mediated efflux and increase ATPase activity of the transporter protein pumps in MCF-7/ADR drug-resistant breast cancer cells [27]. β-Elemene can attenuate SGC7901 resistance to VCR and ADM by decreasing *p*-gp and MRP [28] and attenuates exosome-mediated resistance and metastasis in the multidrug-resistant gastric cancer cell line SGC7901/ADR [29]. In this review, we summarize the mechanisms of cancer drug resistance, as well as the anti-drug-resistant-cancer effects of plant-derived natural products and underlying mechanisms, in order to provide a potent therapeutic strategy for drug-resistant tumors.

## 2. Method

The review process is divided into three main steps: title, abstract, and content screening. This paper reviews previous studies of natural products on cancer drug resistance. Articles were searched in the PubMed, CNKI, Web of Science, X-mol, and Springer databases. The search terms were as follows: plant-derived natural products; cancer; drug resistance; molecular mechanisms. All titles were screened, and 1354 documents were downloaded for abstract screening. After completing the initial screening, 578 articles met the inclusion criteria; finally, the full texts of all 578 retained articles were critically evaluated to avoid duplication, leaving 296 papers to be included in this review.

## 3. Molecular Mechanisms of Drug Resistance in Cancer

**Figure 1:**
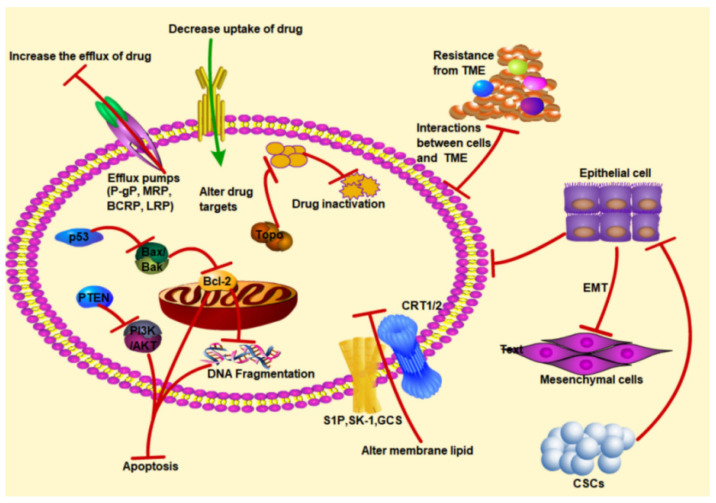
The mechanisms of drug resistance in cancer cells. Two pathways are involved. The intracellular pathways include drug accumulation and absorption reduction, drug target inactivation or alterations, genes involved in apoptosis downregulation, and membrane lipid alteration. In the meantime, the extracellular pathways including EMT, CSCs, and TME also contribute to the development of drug resistance. Abbreviations: P-gP; P-glycoprotein, MRP; multidrug resistance-associated protein, BCRP; breast cancer resistance protein, LRP; lung cancer resistance protein, TME; tumor microenvironment, EMT; epithelial–mesenchymal transition, CSCs; cancer stem cells, Bcl-2; B-cell lymphoma 2, Bax; Bcl-2-associated X protein, Bak; Bcl-2 homologous antago-nist/killer, PTEN; phosphatase and tensin homolog deleted on chromosome 10, PI3K; phosphatidylinositol 3-kinase, AKT; protein kinase B, S1P; sphingosine phosphate, SK-1; sphingosine Kinase-1, GCS; glucosylceramide synthase, CTR1/2; copper transport protein 1/2, Topo; topoisomerase.

Tumor heterogeneity is one of the reasons accounting for the different clinical outcomes of chemotherapy and targeted therapy in cancer patients. Genetic heterogeneity of tumor cells leads to different sensitivities to the various therapeutic agents. Tumor cells that exhibit low sensitivity to chemotherapy drugs bring about drug dissociation and form primary drug resistance [30]. On the other hand, acquired drug resistance develops after long-term exposure of a chemotherapy drug to tumor cells that initially respond to therapy [31]. Many factors that modulate the binding of chemotherapy drugs to tumor cells, DNA damage or apoptosis, cause the emergence of drug resistance [32]. Mechanisms of multiple drug resistance to cancer therapies are complex. Camptothecin (CPT) is extracted mainly from the dove tree, family Hippophae, which is endemic to China. The CPTs, including topotecan (TPT), irinotecan (CPT-11), 10-hydroxycamptothecin (HCPT), etc., have been marketed and widely used in clinical anticancer therapy. Among them, CPT-11, a pre-drug, is converted into the highly active metabolite SN-38 in vivo by the action of carboxylesterases. The current mechanisms of resistance to CPTs include (1) the activation of drug-transporting proteins such as the *p*-gp protein, which excretes intracellular drugs out of the body, (2) alteration of the target of drug action or enhancement of the target’s restorative effect, and (3) inhibition of apoptosis and cell cycle blockade [33]. Chemotherapeutic agents are currently categorized into two groups based on their origins. One is of compounds extracted from plants [34,35], and another is of synthetic compounds [36,37]. According to the mechanism of action, the synthetic ones can be categorized as alkylating agents, antimetabolites, topoisomerase inhibitors, mitotic spindle inhibitors, etc. For example, pyrimidine antagonists (5-fluorouracil (5-FU), gemcitabine, and capecitabine) can be categorized as the antimetabolites, methotrexate, pemetrexed, and pramlintide as antifolates, and hydroxyurea as ribonucleotide reductase inhibitors. These anticancer drugs interfere with important biosynthetic pathways, hinder DNA/RNA synthesis, or cause DNA strand breaks by inhibiting specific enzymes (dihydrofolate reductase, ribonucleotide reductase, and DNA polymerase) or by adulterating DNA with faulty structural analogs of pyrimidines/purines [36,38,39]. Cisplatin is a DNA-intercalating agent that crosslinks DNA, thereby inhibiting RNA transcription and DNA replication activities. If DNA damage is not repaired promptly, cell cycle arrest and apoptosis are triggered [40,41]. Cells can develop resistance to cisplatin through a variety of mechanisms, including alteration of intracellular drug accumulation by inhibition of uptake or enhanced efflux, detoxification of the drug by redox mechanisms, enhanced DNA excision repair or negative regulation of apoptotic signaling [42,43,44,45]. Figure 1 shows the mechanisms of drug resistance in cancer cells, which can be divided into intracellular and extracellular pathways. The intracellular pathway includes drug accumulation and absorption reduction, drug target inactivation or alteration, apoptosis-related genes downregulation, and membrane lipids alteration. At the same time, there are other extracellular factors that contribute to the development of drug resistance, including epithelial–mesenchymal transition (EMT), cancer stem cells (CSCs), and the tumor microenvironment (TME).

### 3.1. Drug Accumulation and Absorption Reduction

The most common mechanism of multidrug resistance is the elimination of drugs from the cell by ATP-dependent efflux pumps, thereby reducing intracellular drug concentrations and giving rise to resistance. ATP-dependent efflux pumps are members of the ATP-binding cassette (ABC) transporter family with sequence and structural homology [46]. It mainly involves P-glycoprotein (P-gP), multidrug resistance-associated protein (MRP) family, breast cancer resistance protein (BCRP), and lung cancer resistance protein (LRP). Among the ABC transporters involved in MDR, P-gP is the most common efflux pump in the plasma membrane [47], as well as a main marker of MDR. Usually, the sensitivity of cancer cells to drugs is enhanced by inhibiting the expression of P-gP [48]. Moreover, it can release the drug into extracellular space and decrease its intracellular concentration, thus inducing drug resistance [49]. It has been reported that P-gP is over-expressed in paclitaxel (PTX)-resistant ovarian cancer cells, and grape seed, procyanidi, is a natural polyphenol supplement that can inhibit the expression of P-gP through downregulating the nuclear factor kappa-light chain-enhancer of activated B cells (NF-κB) activity and the mitogen-activated protein kinase (MAPK)/extracellular signal-regulated kinases (ERK) pathway [50]. MDR is associated with MRP protein if P-gP is normally expressed. MRP is another member of the ABC transporter family, which is encoded by MDR-related protein 1 (MRP1/ABCC1) and also pumps toxic substances out of cells in an ATP-dependent manner [51]. MRP family members are expressed in a variety of cancer cell lines, including lung cancer, bladder cancer, pancreatic cancer, breast cancer, and ovarian cancer [52]. Another ABC transporter protein involved in MDR is breast cancer resistance protein (BCRP/ATP-binding cassette subfamily G member II (ABCG2)), which can transport methotrexate, 7-yethyl-10-hydroxycamptocampin, or tyrosine kinase inhibitors [53]. It is highly expressed in malignant hematopoietic cells and lymphocytes and is closely related to the development of drug resistance [54]. LRP/major vault protein (MVP) is not only localized on the cell membrane but also expressed in the cytoplasm. It is associated with cytoskeletal elements and the nuclear membrane, which can transport drugs from the nucleus to the cytoplasm through vesicle transport [55]. Kitazono et al. reported that sodium butyrate, a differentiation-inducing agent, can increase the levels of expression of LRP mRNA and LRP and confer resistance to adriamycin (ADM) and VP-16 in a human colon carcinoma cell line. Pyrimidine analogues can inhibit the efflux of doxorubicin from nuclei, thereby reversing LRP-mediated resistance [56].

Inhibition of drug absorption may be one of the most effective ways to reduce drug accumulation. Anticancer drugs are transported into the cells through multiple pathways, including diffusion across the plasma membrane, uptake mediated by receptors/transporters, and endocytosis. On the one hand, the development of drug resistance in cancer cells could result from alteration and mutation in the receptors/transporters. Receptor-mediated endocytosis also affects the cellular uptake of certain drugs. It is well known that drug resistance will occur when endocytosis is defective [57]. On the other hand, the biological qualities of cell membranes are also related to the uptake of chemotherapy drugs. Recent molecular dynamics research showed that diffusion of cisplatin (Cis) depends on cell membrane composition. Compared with drug-resistant cancer cells, tumors that are sensitive to cisplatin have higher membrane fluidity [58]. Moreover, the amount of drug accumulation and the mode of passive diffusion into the cell could be regulated through many different factors, including pH, osmotic pressure, temperature, Na+/K+ ATPase, and membrane permeability. 

### 3.2. Inactivation of Drug or Alteration of Drug Target

Multidrug resistance based on non-transport mechanisms mainly reduces the cytotoxicity of drugs by altering enzyme activities or cellular targets [59]. Glutathione S-transferase (GST) is a key enzyme in tumor MDR, which plays an important role in drug detoxification. Glutathione (GSH), an antioxidant, also is a cofactor by the GST enzyme system. It can prevent oxidative stress and maintain a stable oxidation-reductive condition in cells [60]. GSH-synthesizing enzymes are also important in the development of drug resistance. In the process, GSH with alkylating drugs, such as doxorubicin and cyclophosphamide, causes drug resistance in cells through detoxifying bioalkylating agents [61]. A large number of studies have shown that GSH is highly expressed in many drug-resistant cancer cells. GST inhibitors or substrates for GSH conjugation can reverse MDR in tumors through attenuating the detoxification process. Coniferyl ferulate, isolated from Angelica sinensis, is a strong GST inhibitor that reverses doxorubicin (DOX) resistance in endometrial carcinoma [62].

In addition, multidrug-resistant cells overexpressing P-gP also are related to the GSH enzyme system. Substrates, such as trans-chalcone and diethyl maleate, interact with GSH, which can significantly reduce the levels of GSH in many types of tissues [63]. Buthiionine sulfoximine (BSO), an inhibitor of GSH synthesis, has been reported to reverse alkylating drugs resistance through reducing the level of GSH in cells, as well as overcoming resistance caused by MRP [64,65]. Topoisomerase (Topos) play a critical role in DNA replication, transcription, repair, and other processes. They are targets of many antitumor drugs with different action mechanisms and regulate cancer cell drug resistance. Topo I and Topo II are two common types of Topo, which alter the topological state of DNA through DNA strand cleavage and religation. Both are widely used as targets of antitumor drugs. However, Topo II is the most common target of chemotherapy in clinical application. Its inhibitor can directly bind to Topo II or form a DNA–Topo II-drug ternary complex and inhibit the catalytic activity of Topo II, thereby preventing DNA replication and inducing apoptosis [66]. It can cause occurrence of MDR through decreasing levels of Topo II and phosphorylated Topo II and inducing gene mutation or deletion. In the process of special cancer treatment, alteration of a drug target can lead to the failure of treatment. Sometimes, instead of the original target, drug resistant cancer cells generate a mutated target that maintains its activity within the cell but is not inhibited by the drug anymore. Commonly, the therapeutic strategy of blocking the estrogen receptor signaling pathway is effective in estrogen receptor positive breast cancer patients but will lose its effectiveness due to drug resistance during endocrine therapy. It is reported that loss of the therapeutic target is the main reason for endocrine resistance [67].

### 3.3. Inhibition of Apoptosis-Related Gene Expression

Endoplasmic reticulum stress (ERS) resulting from the accumulation of unfolded or misfolded proteins in the endoplasmic reticulum (ER) is involved in regulating the apoptotic process in tumor cells. It will trigger endogenous and exogenous mitochondrial apoptotic signals as ER dysfunction persists in eukaryotic cells. In addition, severe and continuing ERS can lead to cell dysfunction and even cell death [68]. Apoptosis is an active and ordered multistep cell death process and involves two major pathways, intrinsic and extrinsic apoptosis pathways, in human cancer cells. A molecular damage detection system and a pro-apoptotic pathway can determine whether the cells will undergo apoptosis or survive to adapt to the environment [69]. Various proteins are involved in both apoptosis pathways of cells, and alteration of these pathways can lead to the development of drug resistance. p53 is a powerful transcription factor with a tumor suppressor function [70]. Except in stress conditions, such as nitric oxide exposure and oncogenic signaling, p53 protein is inactive in cells with a short half-life. The mutation in a p53 coding gene or deletion occurs in more than 60% of cancers [71]. When p53 is abnormally activated, it will cause uncontrolled proliferation and cell cycles, and the defect in DNA damage repair, in turn, induces cancer drug resistance or further apoptosis [72]. The intrinsic pathway of apoptosis is mediated mainly by mitochondria within the cell, which are regulated by proapoptotic B-cell lymphoma protein 2 (Bcl-2) family proteins [73]. Up to now, 25 Bcl-2 family members have been identified. For example, the BH3-interaction domain death agonist (Bid), Bcl-2-associated X protein (Bax), and Bad are pro-apoptotic proteins; Bcl-x and Bcl-2 exert anti-apoptotic functions. Some chemotherapeutic agents increase the sensitivity of drug-resistant cancer cells through reducing intracellular levels of Bcl-2, resulting in resistant cell apoptosis [74]. It has been reported that overexpression of microRNA-181b (miR-181b) and microRNA-497 (miR-497) can induce apoptosis and reverse drug resistance through decreasing the expression of Bcl-2 in vincristine-resistant gastric cancer and cisplatin-resistant lung cancer [75].

Another important tumor suppressor is the tensin homolog deleted on chromosome 10 (PTEN), which is deficient in a variety of cancers because of mutation, loss of heterozygosity, or epigenetic modification [76]. The PTEN gene encodes a 3,4,5-triphosphate inositol phospholipase, which can negatively regulate PI3K, MAPK, and focal adhesion kinase (FAK) signaling pathways [77]. Loss of PTEN can activate the PI3K/AKT pathway, thereby reducing cell apoptosis and inducing drug resistance [78]. Thidiazuron is a plant growth regulator (phytohormone), which can induce apoptosis of drug resistant MDA-MB-231 cells, which are a triple-negative breast cancer cell line. It can target microRNA-202-5p (miR-202-5p), stimulate the expression of PTEN, and downregulate the PI3K/AKT signaling pathway, thereby inhibiting breast cancer metastasis and progression [79]. In addition, Zhang et al. found that overexpression of microRNA-130b-3p (miR-130b-3p) targets PTEN to promote proliferation, inhibit apoptosis via the Wnt/β-catenin pathway, and significantly enhance the resistance to cisplatin in lung cancer A549 cells [80]. Moreover, autophagy is a double-edged sword for MDR, which participates in the acquired resistance phenotype. It protects cancer cells from the influence of chemotherapy drugs. Meanwhile, it also acts as an executioner that kills MDR cancer cells. High-mobility group box protein 1 (HMGB1) promotes acquired DOX resistance in BEL7402 and SMMC7721 cells by enhancing autophagy. It also can inhibit apoptosis through the adenosine monophosphate-activated protein kinase (AMPK)/mammalian target of the rapamycin (mTOR) signaling pathway [81]. Similarly, Fan et al. demonstrated that peptidylarginine deiminase IV causes MDR in liver cancer cells by inducing autophagy as a protective mechanism [82]. Sun et al. studied two drug-resistant cell lines with high expression of P-gP, MCF-7er, and SK-BR-3er, which exhibit resistance not only to epirubicin but also to PTX and vinorelbine (NVB). PTX and NVB act as a protective mechanism to escape from apoptosis through inducing autophagy [83]. Considering that the relationship of autophagy and MDR is becoming important, the related research is also expanding.

### 3.4. Alteration in Membrane Lipid

Compartmentalization is an important mechanism for drug resistance. Cells are divided into different compartments that sequester drugs in cellular compartments. The pH and composition of lipids and proteins in each compartment affect the localization and accumulation of drugs [84]. Drug targets have different subcellular locations, such as topoisomerase and DNA being in the nucleus, as well as in other organelles, so the distributions of drugs in cells have a critical effect [85]. In many types of cancer cells, there are differences in lipid levels and distribution in the membrane as compared to healthy cells. Although the phospholipid composition is similar, the cholesterol level is significantly lower. It is important to alter the membrane lipid for multidrug resistance [86]. Many studies have shown that upregulated levels of ceramide lead to apoptosis, while sphingosine phosphate (S1P) augmentation inhibits apoptosis [87]. Hun et al. found that sphingomyelin species were significantly increased in 5-fluorouracil (5-Fu)-resistant colorectal cancer (DLD-1/5-FU) cells. The resistance is mainly attributed to ceramide reduction through regulating the activity of acid sphingomyelinase [88]. In addition, compared with tamoxifen (Tam)-sensitive breast cancer cells MCF-7, the levels of ceramide and hexosylceramide are lower in tamoxifen-resistant cells. Meanwhile, endocrine-therapy-resistant cells rely on ceramide kinase and its product, ceramide-1-phosphate, to maintain low levels of ceramide and promote cell survival [89]. Gao et al. established sunitinib-resistant human renal cancer cell lines (786-O, A498, ACHN, and CAKI1). Sphingosine kinase-1 (SK1), which is responsible for the synthesis of sphingosine-1-phosphate, and ERK were activated in sunitinib-resistant cells. Renal cancer cells become sensitive to sunitinib while using the SK1 and ERK inhibitors [90]. Moreover, glucosylceramide synthase (GCS) is increased in drug-resistant cancer cells. GCS and P-gP activities are associated with multidrug-resistance phenotypes. Madigan et al. demonstrated that, compared to sensitive cell lines, oxaliplatin-resistant cells show increased expression of GCS protein and decreased levels of ceramide. Meanwhile, activation of the AKT protein and an increase in anti-apoptotic protein survival are also observed [91]. In addition, inhibition of GCS expression can promote apoptosis of gastric cancer cells and their cisplatin (DDP)-resistant cancer SGC7901/DDP cell through regulating the expression of Bcl2, Bax, and cysteine-aspartic-acid-specific protease-3 (caspase 3) proteins in the apoptotic pathway, and the expression of GCS also affects the multidrug resistance of gastric cancer cells through the MDR-1 pathway [92].

In addition, drug entry into cells is a complex process. Drugs must cross the phospholipid bilayer to reach the intracellular target after contacting the cell membrane. Drug uptake is mainly achieved by passive diffusion and transporters. Copper transport proteins (CTRs), including CTR1 and CTR2, are permeable enzymes located on the cell membrane. Drugs can be transported by CTR1 and enter the cell fluid along the concentration gradient [93]. The abundance of CTR1 in the drug-resistant cell line significantly decreases as compared to the sensitive cell line, and CTR2 hinders cisplatin influx into the cell; the decreased expression of CTR2 promotes cisplatin influx and makes cells sensitive. After knockdown of the 50% gene of CTR2 mRNA, the sensitivity of tumor cells to cisplatin increased 2.6- to 2.9-fold, and overexpression of CTR2 increased cisplatin resistance of tumor cells 2.5-fold. Therefore, decreasing expression of CTR1 will make cells resistant to cisplatin, while the decrease in CTR2 increases the sensitivity to cisplatin [94]. In conclusion, CTR can reduce the influx of cisplatin and form drug resistance through abnormal expression.

### 3.5. EMT

In addition to the intracellular mechanisms mentioned above, the occurrence of cancer cell drug resistance is also influenced by other extracellular factors, including EMT, CSCs, and TME. EMT is a transformation process in which epithelial-derived cells lose their epithelial characteristics and acquire a mesenchymal phenotype [95]. Mesenchymal cells contribute to the development of drug resistance. EMT plays an integral role in the migration and invasion of the epithelial cells and shows an important contribution pathologically to cancer progression [96]. It mainly manifests through decreasing the epithelial marker E-cadherin and increasing mesenchymal markers Vimentin and *N*-cadherin. Moreover, transcription factors play an important role in this process, which can inhibit expression of the epithelial genes, such as Snail and SNAI2 (Slug), and activate expression of the mesenchymal genes, such as Twist1 [97]. More characteristics of EMT appear in cisplatin-resistant cervical cancer cells, which gain metastatic ability as compared with sensitive cells [98]. EMT is recognized as the important factor of chemotherapy resistance in many types of cancer, while inhibition of EMT can enhance chemotherapy sensitivity [99]. It has been reported that iASPP induces cisplatin resistance in HeLa and SiHa cells by stimulating EMT [100]. Qin et al. reported that the expression of vimentin and Snail was increased, but E-cadherin was decreased in the osimertinib (OR)-resistant cell line H1975/OR, and knockdown of Snail can enhance the sensitivity of H1975/OR cells to osimertinib [101]. Fibroblast growth factor receptor 3 (FGFR3) is expressed at high levels in cisplatin-resistant ovarian cancer cells. FGFR3 overexpression enhances the cisplatin resistance in ovarian cancer through elevating the phosphorylation of the epidermal growth factor receptor (EGFR) and further activating the PI3K/AKT signaling pathway [102]. It has been observed that chemotherapy can increase the expression of marker proteins related to EMT, including *N*-cadherin, vimentin, and Slug proteins in drug-resistant human lung squamous carcinoma [103].

In addition, miRNAs are shown to be an important contribution to drug resistance for regulating EMT [104]. Both microRNA-25-3p (miR-25-3p) and microRNA-31-3p (miR-31-3p) reverse the EMT phenotype of cervical cancer cells by directly targeting the Sema4C, increasing the sensitivity of the cells to cisplatin [105]. microRNA-4725-5p (miR-4725-5p) is highly expressed in the plasma of lung cancer patients. It can decrease the protein level of E-cadherin and promote proliferation, migration, and cisplatin resistance in lung cancer cell lines [106]. Overexpression of microRNA-155 (miR-155) inhibits EMT by inducing EGF and reducing the migration and invasion of Caski cervical cancer cells and increases chemical sensitivity to cisplatin [107]. It is suggested that miRNA is a potential mechanism for reversing drug resistance in human basal-like breast cancer through regulating EMT [108]. Moreover, EMT is also ready to help drug-resistant tumor cells to evade immune surveillance. Lou et al. demonstrated that EMT is related to immune action according to the analysis of a large amount of data on lung adenocarcinoma [109]. EMT can promote the expression of many immunosuppressive membrane proteins, including programmed cell death ligand 1(PD-L1), T-cell immunoglobulin, mucin domain containing protein 3 (TIM3), and lymphocyte-activation gene 3 (LAG3). The high expression of these immune checkpoints fosters immune escape. Therefore, the inhibition of EMT is a promising and effective therapeutic approach for drug resistance and metastatic cancer.

### 3.6. Cancer Stem Cells

CSCs are composed of a small group of stem-like cells that have the ability to renew and differentiate into non-stem cells. CSCs can increase the odds of cancer recurrence because of its high tumorigenicity and produce heterogeneous tumors [110]. Normally, CSCs are in a quiescent condition, whereas, once activated, the CSCs will promote tumor cell migration and invasion, thereby inducing the recurrence of disease and the development of drug resistance. High expression of BCRP on cell surfaces can reduce intracellular drug concentration through drug delivery efflux, thereby promoting the occurrence of drug resistance [111]. Generally, conventional chemotherapy only targets non-stem cell tumor cells, resulting in an increasing proportion of stem cells in tumors. CSCs are also a key factor for the induction of drug resistance, regulation of metastasis, and recurrence of malignance [112]. The multidirectional differentiation potential of CSCs makes the tumors formed by single mutant cells heterogeneous, resulting in uneven response to drugs, thereby inducing drug resistance [113]. The transformation zone in the cervical epithelium is rich in stem cells. And persistent high-risk human papillomavirus (HPV) infection always happens in this area, which plays an essential role in cervical cancer development and progression [114]. Regardless of primary or acquired drug resistance in cervical cancer stem cells, CSCs are a key target for chemoresistant cancer treatment [115].

Activity of aldehyde dehydrogenase (ALDH) and CD24-/low/CD44+ are the characteristics of CSC subsets in malignant tumors. Liu et al. reported that, with high ALDH expression, cervical cancer cells are more resistant to cisplatin treatment than cells with low ALDH expression [116]. Overexpression of microRNA-23b (miR-23b) can reduce the expression of ALDH1A1 and disrupt the homeostasis of CSCs, thereby restoring the sensitivity of cervical cancer cells to cisplatin [117]. Cancer cells are in the dormant state with hypometabolism and ameiosis when the environment of tumor cells is terrible. If the living environment conditions are improved, the cells will reenter the proliferative state of active division. CSCs take refuge from anticancer drug therapy through dormancy, but this will cause recurrence of tumors by activating dormant cells [118]. Graciela et al. found that resveratrol in combination with etoposide can induce apoptosis and exert the synergistic anticancer effect through inhibiting the expression of the DNA repair protein RAD51 gene in cervical cancer stem cells [119]. In addition to the classic PI3K/AKT/mTOR signal pathway, the Wnt/β-catenin signaling pathway regulates self-updates and maintains the stemness of CSCs [120]. Once the Wnt/β-catenin signaling pathway is inhibited, cell apoptosis is induced in cervical cancer. Otherwise, the Wnt/β-catenin signaling pathway will promote the development of cervical cancer and the generation of drug resistance if it is over-activated [121].

### 3.7. The Tumor Microenvironment

It has been known that the immune function of the body is closely related to the occurrence and progression of tumors. The TME is considered to be a crucial factor for resistance development in some cancers. It refers to the local steady-state microenvironment of tumor cells in the body, which involves several kinds of multifunctional immune cells and molecules. Cellular components include tumor cells, cancer-associated fibroblasts (CAFs), mesenchymal stromal cells (MSCs), tumor-associated macrophages (TAMs), vascular endothelial cells, and immune inflammatory cells. In addition, non-cellular components include cytokines, growth factors of peptides, an extracellular matrix (ECM), and exosomes [122]. Progression of tumors is also related to TAMs, which are the most abundant immune cells in TME [123]. Interleukin 6 (IL-6) in the TME can induce cancer cell proliferation and angiogenesis and produce drug resistance [124]. Camilla et al. found that inhibition of TAMs can induce interferon (IFN) signal transduction in breast cancer mice, thereby increasing the sensitivity of breast cancer cells to cisplatin [125]. Simone et al. reported that IL-6 is highly expressed in SiHa and HeLa cell lines that are positive for HPV [126].

In addition, CAFs, ECM, exosomes, and inflammation are all associated with drug resistance. There are significant areas of hypoxia in most human tumors, and persistent hypoxia may lead to vicious conversion of the cancer cells. It can promote tumor cell proliferation and metastasis and induce chemotherapy drug resistance. The expression of hypoxia-inducible factor 1 (HIF-1α) will increase under hypoxia, thereby inducing drug resistance through increasing the expression of MDR1 and P-gP [127]. A large amount of evidence suggests that CAFs can alter ECM and mediate inflammation through NF-κB signaling pathways, therefore stimulating tumor proliferation and metastasis [128]. Moreover, development of cancer is accompanied by the occurrence and persistence of immunosuppression. Myeloid-derived suppressor cells, TME, CD4+, and CD8+ T lymphocytes have a significant interactive effect, thereby subsequently attenuating the antitumor immunoreaction. In recent years, miRNAs in exosomes have participated in cell hypoxia and angiogenesis through the changing of oxygen metabolism in the TME, thus affecting cell metastasis [129]. This can also increase the sensitivity to anticancer drugs by inhibiting tumor growth [130].

## 4. Potential Mechanisms of Plant-Derived Natural Products in the Treatment of Drug-Resistant Cancer

As the source of traditional Chinese herbal medicine, China has a wide variety of medicinal plants and extremely abundant species resources. For the past few years, plant-derived natural products have been evaluated as potential anticancer drugs that preferentially kill tumor cells with low toxic effect on normal cells. A variety of plant-derived natural products with antitumor activities have been identified, such as terpenoids [131], alkaloids [132], flavonoids [133], and polyphenols [134]. In addition, plant-derived natural plants can also overcome the drug resistance of tumor cells through multiple molecular and cellular mechanisms, involving the regulation of MDR gene-related proteins, PI3K/AKT signaling pathways, autophagy, NF-κB signaling pathways, and other signaling pathways.

### 4.1. Regulation of MDR Gene-Related Proteins

Multidrug resistance is intimately related to MDR gene-related transmembrane transporters, mainly involving P-gP, MRP, BCRP and LRP [135]. Plant-derived natural products exert antitumor effect mainly through inhibiting the expression or function of related proteins. P-gP, the first human ABC transporter discovered, is highly expressed in drug-resistant tumor cells. It not only inhibits caspase cascade apoptosis but also pumps drugs out of cells through mitochondria, thereby reducing intracellular drug concentration and achieving drug resistance [136]. The natural flavonoid quercetin can reverse the drug resistance in colon cancer cells by inhibiting the activity of the ATP-driven P-gP transporters, enhancing doxorubicin drug accumulation and downregulating the expression of the glutamine transporter solute sarrier family 1, member 5 (SLC1A5) [137]. Rutin, a quercetin glycoside, can restore the sensitivity of human breast cancer cells to cyclophosphamide by nonselective inhibition of the expression of P-gP and BCRP. At the same time, 20 μmol/L rutin can arrest the cell cycle in G2/M phase, and 50 μmol/L rutin can block the G0/G1 phase cell cycle [138]. Zheng et al. reported that another flavonoid compound, myricetin, has robust cytotoxicity on A2780 and OVCAR3 ovarian cancer cells. It can induce apoptosis and enhance the sensitivity of ovarian cancer cells to paclitaxel through downregulating the expression of P-gP [139]. Naringin, which is a dihydroflavonoid compound, in combination with cisplatin can induce apoptosis of cisplatin-resistant human lung cancer A549/DDP cells through increasing the protein levels of Bax and cleaved caspase 3 and decreasing the protein levels of Bcl-2. At the same time, it can also downregulate the protein levels of P-gP, MRP1, *p*-AKT, and CXC chemokine receptor-4 (CXCR4) in a dose-dependent manner [140]. Moreover, Zhu et al. found that naringin can reverse cisplatin resistance in human ovarian cancer SKOV3/DDP cells by decreasing the expression of NF-κB and P-gP [141]. The apigenin can inhibit proliferation of A549/DDP cells and reverse resistance through suppressing the mRNA transcription of MDR1 and the drug extracellular transport function mediated by P-gP, and the reversal index was 2.48 [142]. Silibinin is a flavonolignan extracted from milk thistle (Silybum marianum); it has a synergistic effect with etoposide and doxorubicin, which can induce small-cell lung carcinoma cell apoptosis through decreasing the expression of P-gP [143]. 

In addition, hypericin in combination with vincristine can decrease the expression of P-gP, inhibit the function of the P-gP pump, and enhance the sensitivity of vincristine (VCR)-resistant colon cancer cells HCT8/VCR to vincristine [144]. Epigalcatechin gallate, (-)-Epigallocatechin 3-gallate (EGCG), is a class of polyphenols extracted from green tea, which can reverse the resistance of SiHa/DDP cells to cisplatin with the reversal index of 6.06 through the inhibition of P-gP protein and ABCG2 protein expression in cells [145]. It has been reported that curcumin can induce apoptosis in doxorubicin-resistant chronic myelocytic leukemia K562/DOX cells, and curcumin at 0.5–2 µmol/L reverses doxorubicin resistance with a reversal index of 1.3–9.3. Mechanically, it exerts reversal effects on drug resistance through decreasing the expression of P-gP and S100A8 [146]. Naringenin increased the accumulation of doxorubicin by selectively inhibiting expression of MRP but not of P-gP, thereby inducing the apoptosis of MCF-7/DOX cells [147]. Icaritin, isolated from the medical plant Herba epimedium, was observed to decrease the expression of P-gP, increase the intracellular accumulation of adriamycin, and reverse MDR in the multiple-drug-resistant HepG2/ADR cell line [148]. Emodin is an anthraquinone derivative isolated from the roots of rheumatic palm leaves and has antibacterial properties and anticancer effects. Min et al. revealed that emodin can downregulate the expression of P-gP, competitively inhibit P-gP function, and induce the apoptosis of leukemia K562/ADM cells [149]. It can also increase the sensitivity of pancreatic cancer to gemcitabine by inhibiting the expression of MDR1/P-gP and MRP [150]. Furthermore, Ma et al. confirmed that emodin also significantly induces the apoptosis of cisplatin-resistant ovarian cancer cells COC1/DDP. Co-treatment with emodin and cisplatin can increase the level of reactive oxygen species (ROS) and decrease the expression of MRP1, thereby enhancing the anticancer effect of cisplatin. It also inhibits the tumor growth in vivo by increasing tumor cell apoptosis [151]. Chen et al. demonstrated that β-elemene at 10 μmol/L can reverse cisplatin resistance, with the reverse index being 2.58, and can reduce the expression of resistance-related protein ATP-binding cassette subfamily B member 1 (ABCB1), LRP, and P-gP at a concentration of 20–80 μmol/L [152]. In addition, β-elemene also can reverse the resistance in daunorubicin (DNR)-resistant human leukemia K562/DNR cells, which was associated with an increase in intracellular drug accumulation, the induction of polyadenosine diphosphate ribose polymerase 1 (PARP) cleavage, and a decline in P-gP expression [153]. Furthermore, Daddy et al. co-encapsulated a chemotherapeutic drug of mitoxantrone (MTO) and a P-gP inhibitor of β-elemene (βE) in solid lipid nanoparticles (MTO/βE-SLNs), which can enhance cytotoxicity through increasing the cellular uptake and blockage of intracellular ATP production and P-gP efflux [154]. Chen et al. demonstrated that sinophenine, an alkaloid derived from penicillium stem, reduces the multidrug resistance of human bladder cancer cells to chemotherapy by activating apoptosis-related signaling pathways and downregulating the expression of P-gP [155]. Zuo et al. revealed that matrine can effectively reduce the abundance of the P-gP protein and the activity of the ATP enzyme, control the function of P-gP efflux, and strengthen the concentration of ADM, hence reversing multidrug resistance in BIU-87/ADM cells [156]. Table 1 summarizes their effects and mechanisms on drug-resistant cancer.

### 4.2. Induction of Apoptosis through PI3K/AKT Signaling Pathway

The intrinsic pathway of apoptosis is mediated mainly by mitochondria within the cell, and this pathway is regulated by the proapoptotic protein Bcl-2 family [73]. The PI3K-AKT-mTOR pathway mainly induces cell survival by activating anti-apoptotic factors and inhibiting pro-apoptotic factors. AKT can play an anti-apoptotic role by phosphorylating target proteins in various ways, such as by inhibiting caspase 9 activity to prevent the apoptotic cascade. The PI3K cascade signaling pathway plays a central role in regulating biological processes, such as cell growth, survival, proliferation, and angiogenesis, and is also one of the most common dysregulated pathways in human cancer, leading to abnormal cell proliferation [161]. The PI3K/AKT signaling pathway runs through the occurrence and development of tumors, and the activation of this signaling pathway can activate a variety of downstream signaling pathways to promote the development of tumors and the generation of drug resistance [162]. Baicalin combined with doxorubicin can exert a synergistic effect on doxorubicin-resistant leukemia cells, HL-60/ADM, which induce apoptosis through inhibiting the PI3K/AKT signaling pathway and downregulating the expression of MRP1, LRP, and Bcl-2 [163]. Baicalein can reduce the expression of glycolytic-related proteins (HKII, PDHK1, and LDHA) in a dose-dependent manner, downregulate the expression of the hypoxia-inducible factor (HIF-1α) by inhibiting the PI3K/Akt signaling pathway, thereby reversing the hypoxia-induced drug resistance of colon cancer HCT116 cells [164]. PI3K is negatively regulated by PTEN, which can inhibit the phosphorylation of PI3K and reduce expression of PI3K/AKT signaling. Tanshinone IIA enhances the sensitivity of MCF-7/DOX to doxorubicin by downregulating the expression of P-gP, BCRP, and MRP1 and inhibiting the PTEN/AKT pathway in vitro. It also can enhance the efficacy of chemotherapy and reduce the toxic side effects of chemotherapy in vivo [165]. Resveratrol increases the anti-proliferative activity of K562/ADR cells through increasing the intracellular concentration of bestatin and inhibiting P-gP function and downregulating P-gP expression. It also can potentiate bestatin-induced apoptosis through significantly increasing the activation of caspase 3 and caspase 8 and inhibiting the phosphorylation of AKT and mTOR [166]. Curcumin, a polyphenol compound extracted from the rhizome of the perennial herb turmeric in Zingiberaceae, promotes a reversal of drug-resistant effects in multidrug-resistant L1210/Adr cells. It can downregulate P-gP expression via inhibiting the PI3K/AKT/NF-κB signaling pathway [167]. Nobiletin (5,6,7,8,30,40-hexamethoxyflavone), a citrus methoxyflavone, is a major component of citrus fruits, particularly the peels of oranges (Citrus sinensis). Nobiletin significantly suppresses ABCB1 transporter activity, increases the intracellular accumulation of docetaxel, and decreases the efflux of ABCB1 substrates in A2780/T and A549/T. It also can reverse the resistance of A2780/T and A549/T cells to paclitaxel and doxorubicin through inhibiting the AKT/ERK/Nrf2 pathway and regulating ATP hydrolase activity [168]. Furthermore, nobiletin can decrease the expression of neuroblastoma-derived MYC (MYCN) and MRP1, as well as AKT, glycogen synthase kinase-3β (GSK-3β), and β-catenin, and increase the intracellular accumulation of intracellular adriamycin, thereby reversing adriamycin resistance in A549/ADR cells through suppressing the AKT/GSK-3β/β-catenin/MYCN signaling pathway [169].

**Figure 2:**
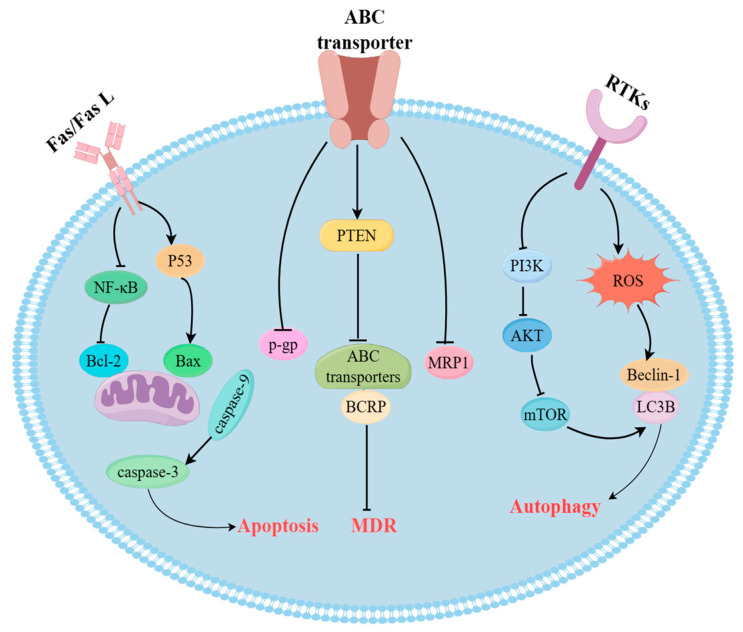
The mechanism of alkaloids and terpenoids for reversing tumor drug resistance. Abbreviations: NF-κB; nuclear factor kappa-B, PTEN; phosphatase and tensin homolog, PI3K-AKT; phosphatidyl-inositol 3-kinase/serine-threonine kinase, mTOR; mammalian target of rapamycin.

Emodin in combination with 5-Fu acts by reversing the resistance of SW480/5-Fu cells to 5-Fu through downregulating the PI3K/AKT signaling pathway. It also can induce apoptosis through attenuating the expression of Bcl-2, increasing the expression of caspase 3 and Bax [170]. Shikonin is a natural naphthoquinone compound isolated from the dry root of Arnebiae Radix. Du et al. confirmed that the drug-resistance index of HeLa/DDP cells was 11.8, and levoshikonin can reverse cisplatin resistance in a dose-dependent manner. In addition, levoshikonin can decrease the expression of Bcl-2 and increase the expression of Bax, cleaved caspase 3, thereby promoting apoptosis and blocking the G0/G1 phase cell cycle of HeLa/DDP cells [171]. 1,4-naphthoquinone can increase the mRNA levels of the H2A histone family, member X (H2AFX), and cause DNA fragmentation, thereby inducing apoptosis and blocking the cell cycle at the G2/M phase in drug-resistant leukemia cell lines [172]. The combination of triptolide and cisplatin can inhibit proliferation of Hela/DDP cells and induce apoptosis and reduce the resistance of Hela/DDP cells to cisplatin, which are attributed to reduction in the expression of Bcl-2, FLIP, and an X-linked inhibitor of apoptosis protein (XIAP) [173]. Yuan et al. demonstrated that cucurbitacin B can downregulate the expression of EGFR, *N*-cadherin, vimentin, *p*-PI3K, *p*-AKT, and *p*-mTOR and upregulate the expression of E-cadherin, thereby inhibiting the EMT of gefitinib (GR)-resistant lung cancer A549-GR cells through suppressing the production of ROS and the PI3K/AKT/mTOR signaling pathway [174]. Li et al. reported that elemene (Figure 2) can increase the mRNA expression of Bax, decrease the expression of Bcl-2, and inhibit the activation of the PI3K/AKT signaling pathway, thus enhancing the sensitivity of A549/DDP cells to DDP [175]. Quercetin can effectively reverse the drug resistance of SiHa/DDP cells to cisplatin, of which the reversal resistance multiple was 4.00. In addition, it can significantly increase the apoptotic rate and decrease the phosphorylation of AKT, mTOR, and p70S6K protein, as well as the expression of P-gP [176]. Luteolin is another natural flavonoid, found in a variety of plants, which has many pharmacological activities. Xu et al. proved that luteolin can reverse the drug resistance of cervical cancer cells to doxorubicin by regulating the activity of the PI3K/AKT signaling pathway. It can increase the expression of PI3K, cleaved caspase 3, decrease the expression of *p*-AKT, *p*-mTOR, and p70S6K in vitro and in vivo, and inhibit the proliferation and metastasis and promote the apoptosis of tumor cells [177]. Matrine is an alkaloid compound extracted from the Chinese herb sophora. It can downregulate the expression of P-gP, MRP1, *p*-AKT, and Bcl-2, upregulate the protein expression of PTEN, Bax, and cleaved caspase 3, and reduce the level of phosphorylated AKT. It can regulate apoptotic factors downstream of the PI3K/AKT signaling pathway, induce apoptosis of doxorubicin-resistant breast cancer cells MCF-7/ADR, and reverse multidrug resistance [178]. Imperatorin is a furanocoumarin compound extracted from the traditional Chinese medicine angelica, which has inhibitory effects on cisplatin-resistant cervical cancer Hela/R cells. It can upregulate the Bim protein and release cytochrome c (CytC) and activate caspase 3, thereby increasing the sensitivity of HeLa/R to cisplatin [179]. Metformin can upregulate the expression of Bax and decrease the expression of Bcl-2 and the mRNA level of AKT1, thereby inhibiting the viability and inducing the apoptosis of the Ishikawa/DDP cell line [180]. Niu et al. successfully established progestin-resistant cells of endometrial cancer and found that metformin can inhibit proliferation of Ishikawa cells and MPA-R-Ishikawa cells in a dose-dependent manner and improve progestin resistance in endometrial cancer cases [181]. Table 2 summarizes the effects and mechanisms on drug-resistant cancer.

### 4.3. Regulation of Autophagy Pathway

Inhibition of the occurrence and development in tumors by autophagy has become a new antitumor therapy. In addition to the PI3K-AKT-mTOR signaling pathway mentioned above, the classical pathway of intracellular autophagy targeted by natural products also involves Beclin-1, p53, and Atg [186]. Many studies have shown that MDR is formed after autophagy, and the level of autophagy in tumor cells of patients with poor prognosis is significantly enhanced, suggesting that the existence of autophagy may promote the development of MDR. Beclin-1 and light chain 3 (LC3) are two key markers of autophagy. Beclin-1 can improve autophagy and induce apoptosis in tumor cells [187]. Isoliquiritigenin, a natural flavonoid extracted from licorice, arrests the cell cycle in the G1 phase and inhibits the growth of human uterine sarcoma cell MES-SA, and it induces autophagy by upregulating the expression of LC3B-II and increases the sensitivity of MES-SA/Dx5 and MES-SA/DX5-R to doxorubicin [188]. Baicalein, another flavonoid compound, can also reverse P-gP-mediated resistance in hepatocellular carcinoma Bel7402/5-Fu through inducing autophagy [189]. Extract of Scutellaria baicalensis in combination with cisplatin can induce autophagy through upregulating the expression of Atg5 and Atg12 in cisplatin-resistant ovarian cancer cell line A2780 (CRC), thereby promoting cell death [190]. In addition, baicalin is a flavonoid derived from the dried root of the labiaceae plant Scutellaria baicalensis Georgi, which can enhance the sensitivity of cervical cancer *C*-33A cells to cisplatin through increasing the mRNA level of autophagy-related genes Beclin-1, Atg5, and Atg12 [191]. Cells undergo swelling and cytosolic vacuolation when autophagy happens, and the formation of autophagosomes and autolysosomes can also be observed. Meanwhile, autophagy shows intact nuclear different from the chromatin condensed in apoptosis [192]. Puerarin is an isoflavone isolated from Pueraria lobata (Willd.) Ohwi. Liu et al. demonstrated that puerarin induces cell cycle arrest and apoptosis in K562/ADR cells and induces autophagy. A large number of autophagosomes are present in the cytoplasm of K562/ADR cells with puerarin treatment, and the expression of LC3-II and beclin-1 are also significantly increased [193]. Hesperetin can inhibit proliferation and autophagy, induce apoptosis, and reverse the resistance of A2780/DDP cells to cisplatin. Mechanically, it increases p53, Bax, and caspase 3, decreases the expression Bcl-2, and regulates the AMPK/mTOR autophagy pathway and p53-dependent signaling pathways [194].

Zhao et al. evaluated the effect of matrine on the doxoruxin-resistant human leukemia K562/ADM cells. The autophagic vacuoles and LC3+ punctate fluorescence by transmission electron microscopy and GFP-LC3 staining increase; matrine upregulates the expression of LC3II and downregulates the expression of p62 in a dose-dependent manner. These results indicated that matrine reversed the resistance of K562/ADM cells to doxorubicin and vincristine by inducing autophagy, and the resistance-reversal multiples were 10.12 and 4.91, respectively [195]. Lycorine effectively inhibited the proliferation of K562 cells and imatinib (IM)-resistant chronic leukemia cells K562/IM, blocked the G0/G1 phase cell cycle, and induced apoptosis in vitro, and the mechanisms underlying encompass the reduction in Bcl-2 expression, stimulation of the caspase 3 pathway, and increase in Bax/Bcl-2 expression. Furthermore, lycorine also can inhibit autophagy, thereby reversing drug resistance in K562/IM cells through downregulating the expression of Beclin-1, Atg5, LC3-II, and P-gP and upregulating the expression of p62 [196]. Cepharanthine can increase the sensitivity of PC-9/IR cells to icotinib (IR) by activating p53-mediated autophagy; it can increase the expression of p53, Beclin-1, LC3-II/LC3-I, and the number of autophagosomes and decrease the ratio of *p*-mTOR/mTOR [197]. Zhou et al. found that β-elemene can increase the sensitivity of lung adenocarcinoma cells SPC-A-1/DDP by reducing the expression of MRP-1 and P-gP, promoting autophagy and increasing the expression of autophagy regulatory protein Beclin-1 [198]. β-elemene can reverse gefitinib resistance in NSCLC cells by inhibiting the cell autophagy process in vitro and in vivo; it inhibits the autophagy flux by preventing autophagic lysosome acidification, resulting in upregulating the expression of SQSTM1 and LC3B-II. Moreover, β-elemene decreases the level of N6-methyladenosine (m6A) methylation of gefitinib-resistant cells [199]. In addition, triptolide induces autophagy in SKOV3/DDP cells in a dose-dependent manner within a certain range through inhibiting the JAK2/STAT3 signaling pathway and, then, inhibit the migration and invasion of cells [200]. The AMPK signaling pathway is one of the important pathways to regulate energy metabolism in cells, which can regulate cellular autophagy. Moreover, naringenin in combination with cisplatin can increase the expression levels of *p*-AMPK/AMPK, Bax, Beclin1, and LC3 II and decrease the expression level of the LC3 I protein, thereby activating autophagy mediated by the AMPK pathway [201]. Kang’ai injection can upregulate the expression of Beclin 1, Atg7, LC3I, LC3II protein, cleaved caspase 3, and the ratio of Bax/Bcl-2, and regulate the interaction between autophagy and apoptosis mediated by Beclin1, which leads to autophagic death and apoptosis of A549/DDP cells [202]. Table 3 summarizes their effects and mechanisms on drug-resistant cancer.

### 4.4. Regulation of NF-κB/MAPK Signaling Pathway

The changes in signaling pathways in tumor cells are closely related to cell proliferation and apoptosis, and intervention against this link is an important chemotherapy strategy for tumor treatment [204]. NF-κB is a family of transcription factor protein complexes, which is mainly composed of p50/p65 heterodimers. It interacts with a variety of cytokines or cytokine receptors and is closely related to the occurrence, proliferation, apoptosis, and invasion of tumor cells. Hesperidin can reduce not only the expression of P-gP but also the activity of NF-κB, thereby reversing the P-gP-induced cisplatin resistance through the NF-κB signaling pathway in cisplatin-resistant human lung cancer A549/DDP cells [205]. Matrine can decrease the expression of ABCB1 by inhibiting the NF-κB signaling pathway and attenuate the doxorubicin resistance of doxorubicin-resistant leukemia K562/ADR cells [206]. Zhong et al. reported that furanodiene causes the apoptosis of doxorubicin-resistant breast cancer cells through activating caspase 8 and accumulating tumor necrosis factor alpha (TNF-α); it also can increase the expression of Bad, caspase 3/7/8, PARP, p65, and IKKα/β and decrease the expression of Bcl-xl [207]. Jiang et al. discovered that triptolide exerted its role in the process of resistance reversal in time- and dose-dependent manners through inhibiting the NF-κB signaling pathway and the transcription and expression of NF-κB-regulated drug-resistant genes, including FLIP, Bcl-2, Bcl-xL, and COX-2 [208]. Schisandrin is the main biologically active component of Schisandra chinensis and belongs to biphenyl cyclooctene-type lignans. It can specifically reverse P-gP-mediated doxorubicin resistance in MCF-7/DOX cells by blocking P-gP, NF-κB, and Stat3 signaling; it can enhance the accumulation of intracellular doxorubicin and increase the DOX-induced cleavage of caspase 9 and PARP1 levels [209].

**Figure 3:**
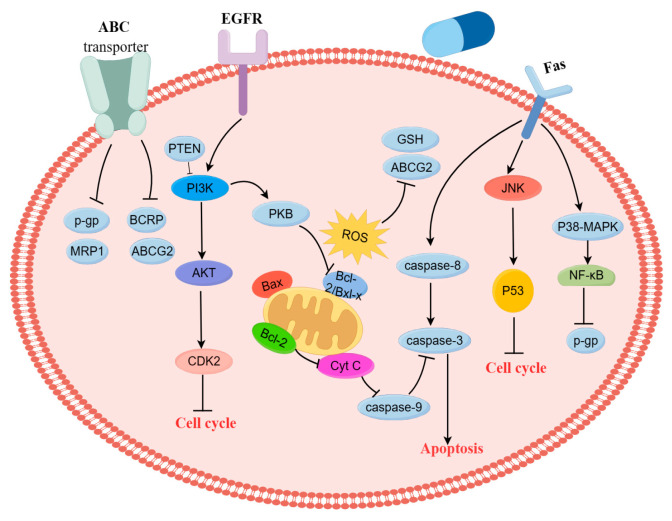
The mechanisms of polyphenols and flavonoids for reversing tumor drug resistance. Abbreviations: PKB; protein kinase B, PTEN; phosphatase and tensin homolog, PI3K-AKT; phosphatidyl-inositol 3-kinase/serine-threonine kinase, CytC; cytochrome C.

The mitogen-activated protein kinase (MAPK) signaling pathway plays an important physiological role in eukaryotic cells [210], mainly including p38, JNK, and ERK pathways, which participate in many bioprocesses, such as cell proliferation, differentiation, apoptosis, and so on [211]. Among them, the p38 pathway can promote cell apoptosis by activating c-Jun and increasing TNF-α expression. Meanwhile, studies have shown that activation of the p38 pathway can lead to cell cycle arrest and apoptosis [212]. Activation of JNK can upregulate the expression of pro-apoptotic proteins and activate caspase 3 [213], while inhibition of ERK can promote cell apoptosis and inhibit the proliferation of tumor cells [214]. p38 MAPK can be activated by upstream signals involved in the control of various stress responses and DNA damage—for example, cell cycle arrest, mitochondrial damage, and apoptosis [215]. In tumor cells, the p53 tumor suppressor gene is inactivated, which may cause cell cycle arrest through p21 deactivation. Resveratrol can downregulate the expression of p38, p65, MDR1, and P-gP via inhibiting the NF-κB and p38 MAPK signaling pathways, thereby reversing the resistance of U2OS/ADR cells to adriamycin [216]. In addition, rosmarinic acid can induce the death of cisplatin-resistant non-small-cell lung cancer cell A549/DDP. It can upregulate the expression of p53, p21, and JNK, inhibit P-gP expression, and activate the MAPK signaling pathway [217]. Xu et al. demonstrated that the combination of baicalin and cisplatin can reduce the expression of MAPK2 and *p*-AKT in a dose-dependent manner and induce apoptosis through the AKT/mTOR pathway in A549/DDP cells [218]. Curcumin can reverse the multidrug resistance of esophageal carcinoma cells through decreasing the expression of p38MAPK, *p*-p38MAPK, ERCC1, and P-gP [219]. Moreover, Cao et al. confirmed that HepG2/ADM cells were moderately resistant to adriamycin with a resistance index of 6.81, and 5 μg/mL curcumin obtained a reversal index of 1.49 for the adriamycin resistance of HepG2/ADM cells (Figure 3). Curcumin can increase the protein expression level of the phosphorylated p38MAPK and inhibit the proliferation of HepG2/ADM in vitro [220]. Paeonol can inhibit the proliferation, migration, invasion, and glycolysis and promote the apoptosis of apatinib (AP)-resistant gastric cancer cells through the linc00665/miR-665/MAPK1 axis. It decreases linc00665 and MAPK1 expressions in gastric cancer cells but increases the expression of miR-665 in vitro. Additionally, paeonol can significantly inhibit lung metastasis in the tumor xenograft mice model [221]. Rotundioic acid participates in the regulation of the MAPK signaling pathway by upregulating the expression levels of *p*-p38 and *p*-ERK1/2 in K562/ADM cells, thus inhibiting MDR1 expression at the transcription and translation levels and finally reversing the multidrug resistance effect of leukemic cells [222]. Isolineralactone is a sesquiterpene compound extracted from Lindera aggregata, which can decrease cell viability and colony formation in both colorectal cancer oxaliplatin (Ox)-sensitive (HCT116 and HT29) and Ox-resistant (OxR) (HCT116-OxR and HT29-OxR) cells. It also blocks the cell cycle at the G2/M phase, induces ROS generation, activates the phosphorylation of JNK/p38 MAPK and multi-caspase, and depolarizes the mitochondrial membrane potential (MMP), which eventually trigger apoptotic cell death [223]. Tetrandrine in combination with vincristine can decrease the expression of *p*-ERK, increase the expression of *p*-P38 and *p*-JNK, activate the degradation of PARP and cleavage of caspase 3/9 via the MAPK signaling pathway, thereby inhibiting proliferation and inducing apoptosis in SGC-7901/VCR cells [224]. Table 4 summarizes their effects and mechanisms on drug-resistant cancer.

### 4.5. Blocking Cancer Cells Cycle

Uncontrolled cell division is an essential factor in the development of cancer. If stagnation occurs, the normal growth and gathering of the cells are hindered, which inhibits proliferation [226]. Cyclins and their cognate cyclin-dependent protein kinases (CDKs) are the necessary components required for traversing the cell division cycle, with controlling balance of all stages in cell cycle [227]. CDK2/4/6, cyclin D1, and cyclin E are the major regulatory proteins in the G1 phase, and the reduction in cyclin B1 and CDK1/2 activity is a key marker of cycle arrest in the G2/M phase [228,229]. Shikonin in combination with cisplatin arrests the cell cycle at the G1/S phase and induces the apoptosis of cisplatin-resistant SKOV3/DDP cells. It also can increase the protein levels of cyclin D1, CDK2, *p*-Rb, and Bcl-2, decrease the expression of p18, Bax, and cleaved caspase 3, and reverse the cisplatin resistance of ovarian cancer SKOV3/DDP cells [230]. Plumbagin (5-hydroxy2-methyl-1,4-naphthoquinone) is another naphthoquinone extracted from the roots of Plumbago zeylanica L, which has significant antitumor activity in vitro. It is able to induce apoptosis and increase ROS production and to arrest the cell cycle in the S phase and G2/M phase through inhibiting the expression of hyperphosphorylated retinoblastoma protein (p-Rb) in both sensitive (A431wt) and cisplatin-resistant (A431/Pt) human cervix squamous carcinoma cell lines [231]. Wang et al. demonstrated that tetramethylpyrazine can effectively reverse the MDR of Pumc-91/ADM and T24/DDP cells through inhibiting the expression of MRP1, GST, and Bcl-2 and increasing the expression of TPOP-II in a dose-dependent manner. In addition, it leads to the blockade of the G1 phase in Pumc-91/ADM and T24/DDP cells [232]. Homoharringtonine inhibits the proliferation of vemurafenib-resistant melanoma cells A375R in a dose- and time-dependent manner, with IC50 values of 18.57 ng/mL, 9.88 ng/mL, and 9.23 ng/mL at 24 h, 48 h, and 72 h, respectively. It reduces the protein levels of IRS4, PI3K, *p*-AKT, and *p*-ERK and induces G0/G1 cell-cycle arrest through reducing the expression of cyclin E1 and CDK2 [233]. 

Yang et al. synthesized a triptolide succinate monoester by catalyzing triptolide and succinic anhydride under DMAP/pyridine, which has a significant antitumor effect in numerous types of cancer. This compound showed selective sensitivity for A549/DDP, A549, and MCF-7 cells with IC50 values of 33.32, 4.31, and 16.08 µmol/L, respectively. It also presented high cytotoxic activity to cisplatin-resistant lung cancer cell A549/DDP and promoted apoptosis and blocked the G2/M phase cell cycle. The mechanism may be related to the inhibition of the interaction between the murine double minute gene 2 (MDM2) protein and p53 [234]. Kamebakaurin significantly suppressed the proliferation of HepG2/ADM cells, and the IC50 value at 24 h was 62.96 µmol/L. It can induce apoptosis and cell cycle arrest in the G2 phase in a dose-dependent manner and also suppress its migration ability through inhibiting the expression of MDR1 and the PTEN-AKT pathway [235]. Ginsenoside Rh2 can decrease the expression of MRP1, MDR1, LRP, and GST, block the G0/G1 phase cell cycle, induce apoptosis, and suppress the migration process and EMT process, thereby effectively reversing the drug resistance of human colorectal carcinoma cell (LoVo/5-Fu and HCT-8/5-Fu) to 5-Fu [236]. In addition, curcumin can reverse 5-Fu resistance and nicotinamide *N*-methyltransferase (NNMT)-induced cell proliferation through ROS generation and cell cycle arrest; it can inhibit the expression of NNMT and *p*-STAT3 in NNMT-related resistance in colorectal cancer cell SW480/NNMT [237]. The combination of isoxanthohumol and cisplatin can inhibit A549/DDP cell proliferation, arrest the cell cycle in the G0/G1 phase, and promote apoptosis, and the mechanism is related to the inhibition of the expression of resistant proteins P-gP, LRP, LRP, and MRP, decreasing the expression of PI3K and *p*-AKT and suppressing the activity of PI3K/AKT signaling [238]. Metformin can reverse the oxaliplatin-resistant human gastric cancer SGC-7901/L-OHP to oxaliplatin, and the reversal index was 3.28 times. Metformin in combination with oxaliplatin blocks the G1 phase cell cycle through decreasing the levels of cyclin D1 and promoting the apoptosis of SGC-7901/L-OHP cells [239]. A Buzhong Yiqi decoction can inhibit cell migration and arrest the G1/S phase cell cycle in cisplatin-resistant human gastric cancer cell lines SGC-7901/CDDP. It also can induce cell apoptosis and enhance the chemosensitivity of SGC-7901/CDDP to oxaliplatin and improve the effect of chemotherapy through decreasing the expression of Bcl-2 and NF-κB cytokines [240]. Digitoxin is a natural cardiac glycoside derived from digitalis, which can significantly suppress the proliferation of adriamycin-resistant human liver cancer cells HepG2/ADM in dose- and time-dependent manners, with IC50 values of 132.65, 52.29, and 9.13 nmol/L at 24 h, 48 h, and 72 h, respectively. It induced G2/M cell cycle arrest via the serine/threonine-protein kinase ATR (ATR)-serine/threonine-protein kinase Chk2 (CHK2)-M-phase inducer phosphatase 3 (CDC25C) signaling pathway in HepG2/ADM cells and induced mitochondrial apoptosis through increasing the ratio of Bax/Bcl-2 and cleaved caspase 3/caspase 3, as well as cleaved caspase 9/caspase 9 [241]. Table 5 summarizes their effects and mechanisms on drug-resistant cancer.

### 4.6. Inhibiting Growth of Cancer Stem Cells

CSCs are cancer cell subsets with self-renewal and multidirectional differentiation characteristics. They are considered to be the origin of cancer and the basis of the cancer malignant phenotype and are conducive to tumor recurrence, drug resistance, and metastasis after treatment and are targets of cancer therapy [242]. Luteolin can inhibit the expression of sex-determining region Y-box2 (SOX2) and affect the characteristics of stem cells by downregulating the PI3K/AKT signaling pathway. It also can downregulate the expression of Slug, *N*-cadherin, and vimentin, upregulate the expression of E-cadherin, and inhibit EMT and migration, thereby inducing the apoptosis of paclitaxel-resistant esophageal cancer cells TE-1/PTX and EC109/PTX [243]. Furthermore, Zhao et al. found that luteolin can reduce the phosphorylation of AKT (S473) and UBR5, inhibit the PI3K/AKT pathway, and decrease the expression of SOX2, significantly attenuating the stem cell properties of paclitaxel-resistant cancer cells TE-1/PTX and EC109/PTX [244]. Wang et al. found that the expression of CD44+/CD24-/low and *p*-AKT in MCF-7/DOX cells is higher than MCF-7 cells; instead, the expression of PTEN is decreased. Quercetin in combination with doxorubicin increases the expression of PTEN and downregulates the expression of *p*-AKT in doxorubicin-resistant human breast cancer MCF-7/DOX. It can effectively reverse the drug resistance of MCF-7/dox cells to doxorubicin through killing breast cancer stem cells and regulating the PTEN/AKT signaling pathway [245]. Matrine significantly reduces the resistance gene expression of ABCB1 and inhibits the protein expression of P-gP, *p*-PI3K, *p*-AKT, and *p*-mTOR, vis the PI3K/AKT/mTOR signaling pathway, thereby reversing chemoresistance in HCT116/5-Fu cells and promoting apoptosis [246]. Manogaran et al. developed a colorectal CSC by using pulse exposure of cisplatin to parental HCT-15 cells. Neferine, isoliensinine, and cisplatin exhibited a stronger cytotoxic activity against CSCs, with IC50 values of 6.5 μmol/L for neferine, 12.5 μmol/L for isoliensinine, and 120 μmol/L for cisplatin. Isoliensinine can induce the generation of ROS and block the sub G0 phase cell cycle, alleviate the expression of ERCC1, and decrease the cell survival protein expression (PI3K/pAKT/mTOR) and BCl-2, upregulating the expression of Bax, cytochrome c, caspase 3, and PARP cleavage, thereby activating mitochondria-mediated apoptosis in CSCs [247]. Moreover, ursolic acid inhibited tumor growth in nude mice transplanted with ovarian cancer stem cells in a dose-dependent manner, decreased the expression of ABCG2, promoted the apoptosis of CSCs, and improved the ability of cisplatin-induced apoptosis [248]. Li et al. successfully established 5-fluorouracil-resistant and oxaliplatin (OX)-resistant colon cancer cell lines HCT116/OX and HCT8/5-Fu, and the stem-cell-related genes were upregulated, such as CD133, CD24, ALDH1, OCT-4, SOX-2, and Nanog1. Curcumin can effectively inhibit the proliferation of drug-resistant cell lines and reduce the expression of CD133, ALDH1, and CD24 [249]. The Yi-qi-hua-yu-jie-du decoction can decrease the expression of MDR1 and MRP1, suppress the activation of the PI3K/AKT/Nrf2 signaling pathway, induce apoptosis, and reverse the drug resistance of BGC823/5-Fu-CSCs [250].

The Hedgehog signaling pathway is a widely conserved signaling pathway in mammals, which has been found to be involved in many physiological processes, such as the processes of cell division, differentiation, regeneration, and epithelial–mesenchymal transition (EMT) [251]. Studies have shown that an abnormally activated Hedgehog signaling pathway promotes the growth and self-renewal of colon cancer stem cells [252]. CSCs are believed to be related to the drug resistance of tumors and ultimately promote cancer recurrence [253]. Genistein significantly inhibits tumor sphere formation in SMMC-7721 cells and preferentially inhibits cell proliferation activity. It can also decrease the protein expression levels of CD133, CD44, glioma-associated oncogene homoglog 2 (Gli1), and ABCG2, and block the Hedgehog signaling pathway, thereby effectively reversing the resistance to fluorouracil in multidrug-resistant liver cancer cell SMMC-7721 [254]. Norcantharidin is a derivative of cantharidin, which can downregulate the expression of cancer stem cell markers Nanog, Sox2, and Bmi1, upregulate the expression of Hedgehog signaling pathway related-molecule Ptch-1, and downregulate the expression of Shh. It can inhibit the proliferation of cisplatin-resistant ovarian cancer cell A2780Cis and paclitaxel-resistant ovarian cancer cell SKOV3Pac through inducing the apoptosis of tumor stem cells and regulating the Hedgehog signaling pathway [255]. In addition to the classical PI3K-Akt-mTOR signaling pathway, there is also the Wnt-β-catenin signaling pathway, which regulates self-renewal and stemness maintenance in CSC [256]. In cervical cancer, if the Wnt signaling pathway is inhibited, it induces apoptosis and inhibits tumor growth, but if it is over-activated, it promotes the development of cervical cancer and the generation of drug resistance [121]. Curcumin inhibits the expression of Wnt2, β-catenin, and vimentin, increases the expression of GSK-3β, and inhibits the EMT, thereby enhancing the sensitivity of 5-fuorouracil-resistant gastric cancer cells BGC-823/5-Fu to cisplatin [257]. Table 6 summarizes their effects and mechanisms on drug-resistant cancer.

### 4.7. Other Signaling Pathways

Current studies have also found that the activities of Chinese herbs against drug-resistant cancer cells are mediated by other different pathways, including the Notch signaling pathway, EMT, JAT2/STAT3, etc. The Notch signaling pathway plays an important role in a variety of cellular processes, including proliferation, differentiation, apoptosis, and stem cell maintenance. Abnormal Notch signaling leads to a variety of diseases and cancers. Baicalin can inhibit the tumorigenesis of human leukemia cell K562 by regulating the endogenous Notch signaling pathway [258]. Sinomenine is an alkaloid extracted from the traditional Chinese medicine caulis sinomenii, which has significant antitumor effect. It can decrease the expression of Notch1, STAT3, and HES1 and inhibit the Notch1/STAT3 signaling pathway, thereby reducing chemotherapy resistance and promoting the apoptosis of cisplatin-resistant cervical cancer cells [259]. Studies have found that EMT plays a key role in the process of drug resistance of tumor cells [260]. Studies have found that epithelial cells with mesenchymal changes have stronger mesenchymal phenotypes such as migration, invasion, and anti-apoptosis, which are also among the main reasons for the invasion, metastasis, and drug resistance of epithelial-derived tumor cells [261]. Tumor cells with interstitial characteristics are also characterized by primary drug resistance. EMT can be observed in drug-resistant tumor cells [262]. Quercetin can increase the expression of miR-101 and decrease the expression of the enhancer of zeste homolog 2 (EZH2), TGF-β1, and *p*-SMAD Family Member 4 (SMAD4)/SMAD4, thereby inhibiting EMT and reducing resistance of the gemcitabine (Gb)-resistant NSCLC cell A549/Gb [263]. Astragalus polysaccharide combined with cisplatin can reduce tumor weight and the metastatic nodules of the lung tumor in BALB/c nude mice through inhibiting the EMT in vivo; it can decrease the expression of α-catenin and elevate the expression of *N*-cadherin, LRP, MRP, and P-gP [264]. Matrine can significantly inhibit the proliferation and metastasis of A549/DDP cells and reverse cisplatin resistance through upregulating the expression levels of E-cadherin, downregulating the expression levels of vimentin, Slug, and *p*-p65 protein [265]. 

Celastrol is a plant-derived triterpene that can significantly reduce the viability of cisplatin-resistant nasopharyngeal carcinoma cells Cis-039 and Cis-BM in dose- and time-dependent manners. It can block the G2/M phase cell cycle, activate caspase, and increase the phosphorylation of MAPK pathway proteins, such as p38 and ERK1/2, thereby inducing Cis-039 and Cis-BM cell apoptosis through intrinsic and extrinsic apoptotic pathways [266]. Oridonin can reverse fulvestrant (Ful)-resistant breast cancer MCF/7-Ful; the resistance index is 4.2. Oridonin in combination with fulvestrant increases the expression of γ-H2AX, Bax, caspase 3, caspase 9, and the ratio of LC3II/I, decreases the expression of Bcl-2 and cyclin D1, and induces generation of DNA damage, thereby giving rise to apoptosis and blocking the cell cycle in the G0/G1 phase [267]. Yao et al. confirmed that β-elemene inhibits the proliferation and decreases the cytoplasmic glutathione levels and the expression of P-gP in a time- and dose-dependent manner; it decreases mitochondrial membrane potential and increases concentration of intracellular ROS and accumulation of rhodamine-123, thus enhancing the sensitivity of A549/DDP cells to cisplatin, and induces apoptosis, leading to reverse drug resistance [268]. Furthermore, the JAK2/STAT3 signaling pathway is involved in the proliferation, differentiation, development, and metastasis of tumor cells. It is an important factor in the pathogenesis of various malignancies and is closely related to microangiogenesis, whose activation can effectively upregulate the expression of angiogenic factors such as vascular endothelial growth factor (VEGF) and basic fibroblast growth factor (bFGF), thus is closely related to the proliferation of tumor cells. It also can activate the transcription of Bcl-2 and Bcl-xl and regulate cell apoptosis. β-elemene also can inhibit the drug resistance of lung cancer cells A549/Taxol to paclitaxel by inhibiting the JAK2/STAT3 signaling pathway, inhibiting tumor cell proliferation, and inducing apoptosis; it can decrease the expression of JAK2, STAT3, *p*-STAT3, and Bcl-2 and increase the expression of Bax and caspase 3 [269]. Shilnikova K et al. demonstrated that shikonin induces mitochondria-mediated apoptosis and attenuates the EMT in A2780-CR cells; it suppresses the level of Bcl-2 and increases the expression of Bax and the cleavage of caspase 9 and caspase 3 [270]. In addition, Wang et al. demonstrated that shikonin, as a specific pyruvate kinase M2 (PKM2) inhibitor, has a significant synergistic effect with cisplation, which can induce necroptosis of cisplation-resistant bladder cancer cell T24R. It can upregulate the phosphorylation of RIP3, enhance the expression of the P53 upregulated modulator of apoptosis (PUMA), Bax, reduce the expression of Bcl-2, and induce T24R cell death [271]. Table 7 summarizes their effects and mechanisms on drug-resistant cancer.

## 5. Synergistic Effects and Combination Therapies

Currently, the development of anticancer drugs is the mainstay of cancer treatment. However, there are many challenges associated with the use of single-agent therapy, including the emergence of drug-related side effects and drug resistance [276]. As a result, there has been an increasing interest in the strategies of combination therapies, which attempt to capitalize on synergistic effects by combining multiple drugs to treat multiple targets, subgroups, or diseases simultaneously [277]. Combination therapy significantly improves effectiveness compared to the traditional single-drug, single-target treatment paradigm. It has been found that EGCG is able to sensitize chemotherapy-resistant cancer cells, and it can act synergistically with various anticancer drugs in cancer treatment, such as cisplatin, oxaliplatin, temozolomide, resveratrol, doxorubicin, vardenafil, curcumin, erlotinib, and others [278,279,280,281,282,283,284,285]. In human ovarian cancer SKOV3 and OVCAR3 cells, EGCG enhances sensitivity to cisplatin by upregulating copper transporter protein 1 (CTR1), leading to the accumulation of intracellular cisplatin and cisplatin-DNA adducts, and the combination of EGCG and cisplatin inhibits tumor growth in OVCAR3 xenograft mice [278]. In addition, the combination of low concentrations of EGCG and curcumin significantly inhibits cell and tumor growth in human non-small-cell lung cancer (NSCLC) A549 and NCI-H460 cells, as well as in A549 xenograft mice [279]. Similarly, the combination of dihydroartemisinin (DHA) and gemcitabine had a strong synergistic effect on the loss of mitochondrial membrane potential and induction of apoptosis in human non-small-cell lung cancer (NSCLC) A549 cells [286]. DHA also enhances the anticancer activity of the chemotherapeutic drug cisplatin in cisplatin-resistant ovarian cancer cells [287]. In addition, ursolic acid (UA) in combination with doxorubicin enhances cellular uptake of adriamycin and reverses multidrug resistance (MDR) in human breast cancer MCF-7/ADR cells [288]. Tanshinone IIA is an effective drug to inhibit DOX resistance in gastric cancer by inducing cell cycle blockade. Combination with DOX enhances apoptosis and triggers autophagic cell death, increases the expression of p53, Bax, and LC3BII, and decreases the expression of Bcl-2 and p62 [289,290].

In addition to individualized compounds, a number of herbal compound components have been shown to have antitumor functions, and some are already in clinical trials. Shengmai injection is a traditional Chinese medicine injection processed using modern pharmaceutical technology, and its active ingredients mainly include ginsenoside, oligosaponin, and tretinoin. Studies have shown that Shengmai injection has the efficacy of potentiating chemotherapy [291], and the use of Shengmai injection in combination with chemotherapeutic drugs can suppress the growth of transplanted tumors of VCR-resistant gastric cancer cells SGC7901 in nude mice, increase the lethality of chemotherapeutic drugs on gastric cancer cells, and accelerate the apoptosis of tumor cells [292]. The main ingredients in Yiqi Jianpi Huaji Tang include Astragalus, Radix et Rhizoma Ginseng, Rhizoma Atractylodis Macrocephalae, Radix Paeoniae Alba, Radix Paeoniae Alba, Citrus aurantium, Fructus Lycii, pine root, and Salvia divinorum. Some scholars have pointed out that the combination of Yichijianpi Huaji Tang combined with 5-Fu can increase apoptosis and block the cell cycle in the S phase. Yiqi Jianpi Huaji Tang increased the sensitivity of SGC7901/VCR cells to chemotherapy by decreasing the expression of MDR1/P-gp, MRP, TUBB3, and STMN1 [293].

## 6. Clinical and Preclinical Research

Clinical trials can confirm or reveal a drug’s action, adverse effects, and pharmacokinetics. Tetrandrine is an isoquinoline alkaloid, which has been shown to significantly reduce the expression of P-gp and LRP and attenuate MDR. Clinical studies have shown that patients have a better toleration of tetrandrine and its use as an adjuvant to chemotherapy [294]. Derivatives of tetrandrine are effective in reversing MDR by inhibiting P-gp transporter and ATPase activity. The mechanism of this action is related to the blockade of the MEK-ERK (mitogen-activated protein kinase-extracellular signal-regulated protein kinase) signaling pathway [295]. 7,3′,4′-trihydroxyisoflavone (THIF) is the major metabolite of daidzein. It was found that adriamycin combined with THIF had better clinical efficacy in cervical cancer. THIF negatively regulates MDR1 by controlling transcription factors and then generates new MDRs [296]. In vivo, β-elemene significantly enhanced the antitumor activity of DOX and increased caspase 3 protein expression in nude mice bearing SGC7901/ADR xenografts [182,297]. Shengmai injection combined with chemotherapy can limit the growth of transplanted tumors of VCR-resistant gastric cancer cells SGC7901 in nude mice, increase the lethality of chemotherapeutic drugs on gastric cancer cells, and accelerate tumor cell apoptosis [292]. Qian et al. demonstrated that berberine can synergistically enhance the inhibitory effect of doxorubicin on tumor cell proliferation in MCF-7/DoxFluc, and the optimal combination ratio was Ber/DOX = 2:1 through using a luciferase reporter assay system combined with the bioluminescence imaging technology. In addition, it can significantly downregulate the expression of P-gp/ABCB 1 and MRP 1/ABCC1 in vivo, reduce the efflux of DOX, increase the uptake of DOX in tumor tissues, and improve the concentration and retention rate of DOX in tumor cells [159]

## 7. Conclusions

Malignant cancer is one of the diseases with the highest mortality rate globally, which seriously endangers human health of the world’s residents, and the number of cases is increasing with each passing year. A major reason for treatment failure in cancer patients is the resistance of primary or acquired chemotherapeutic agents. We have described the most effective drug-resistant mechanisms in this review, including intracellular and extracellular pathways. The intracellular pathway includes reducing drug accumulation and absorption, inactivating or altering drug targets, inhibiting the expression of apoptosis-related genes, and altering membrane lipids. Meanwhile, there are other extracellular factors that contribute to the development of drug resistance, including EMT, CSCs, and tumor microenvironments. In addition, genetic mutations, epigenetic alterations including DNA methylation, histone alterations, and miRNAs, can modulate the development of multidrug resistance [298]. Using high doses of drugs to overcome drug resistance is ineffective and toxic; therefore, there is an urgent need to develop drugs with a better safety profiles and higher efficacy for drug-resistant cancer treatment. 

**Figure 4:**
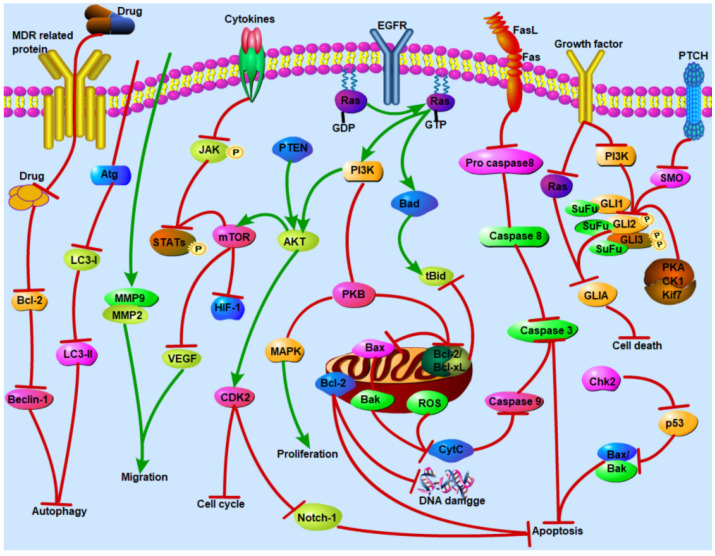
Overview of signaling pathways against resistant cancer. Plant-derived natural products can achieve practical anti-resistant-cancer effects through multiple mechanisms, including induction of apoptosis and autophagy, blocked cell cycle, and inhibition of cell proliferation. For example, they could exert anticancer effects through reducing the expression of MDR-related protein and increasing the concentration of the drug. Moreover, they could induce autophagy by increasing the expression of Beclin-1. They can inhibit the expression of Atg and subsequently increase the ratio of LC3-II/LC3-I. Furthermore, they can promote apoptosis through activation of STAT3 and Notch-1, upregulating the expression of Bax, decreasing the expression of Bcl-xL and Bcl-2, and suppressing the PI3K/AKT/mTOR signaling pathway and caspase cascade reaction. In addition, they can reduce the transcriptional activities of MMP-2 and MMP9 to inhibit metastasis; they also suppress migration through reducing the expression of VEGF. In the next place, natural plants can reduce cancer cell proliferation by binding PI3K and subsequently regulating the MAPK signaling pathway. In addition, they can induce cell death through the Hedgehog signaling pathway. Moreover, they can block the cell cycle by inhibiting the levels of cyclin-dependent kinases. Abbreviations: JAK; Janus kinase, GDP; guanosine diphosphate, GTP; guanosine triphosphate, CytC; cytochrome c, Fas; factor-associated suicide, FasL; Fas ligand.

In recent years, plant-derived natural products and their secondary metabolites have been found to possess characteristics of abundant products, low toxicity and side effects, diverse biologic activities, and high content of active ingredients. They have been considered to be the most promising candidates for oncology therapies. Our study reviewed that natural products have significantly antitumor effects on cancer, which showed possible benefits in treating cancer patients through numerous mechanisms, such as regulation of MDR-related genes, inhibition of the PI3K/AKT signaling pathway, induction of autophagy, and regulation of cell cycle arrest (Figure 4). Natural products with plant origins have many advantages such as abundant resources, low toxicity, and diverse targets with various molecular mechanisms. The diversity of molecular structure and unique action mode of biological activity make them play an important role in the development of candidates for drug-resistant cancer treatment. Recent studies have largely expanded the scope of candidate compounds to all natural products, including herbal extracts, herbal compound preparations, and derivatives, with a view to finding new directions for highly effective tumor multidrug-resistance therapy. However, due to the known problems, such as extraction difficulties, poor solubility, poor permeability, low bioavailability, unstable biological environment, and extensive metabolism in the drug delivery system of natural products, in addition, certain natural plants may have drug–drug interactions with chemotherapeutic drugs and effects on the body. However, due to the lack of therapeutic targets and therapeutic strategies, more research on natural products is needed to characterize their mechanisms of action and possible roles in MDR therapy. Researchers have now explored various methods and approaches to overcome drug resistance. Particles, nanomedicines, and gene editing techniques such as CRISPR/Cas9 have been discovered to overcome multidrug resistance in tumor cells [298].

Resistance may arise due to alterations in the stroma and tumor microenvironment, and anticancer drugs may generate resistance by altering the target and drug efflux pumps, increasing cell tolerance to apoptosis, and accelerating tumor cell proliferation [299]. In the study of drug-resistance mechanisms, most of the current studies have focused on the classical mechanisms of the MDR gene and the P-gp encoded by the MDR gene, while few studies have focused on non-classical mechanisms such as GST-π, Topo II, DNA damage repair, and tumor stem cells. In contrast, these mechanisms are rarely explored and relatively understudied in depth. In addition, most studies on cancer drug-resistance mechanisms have focused on the cellular level and experimental animals. However, the mechanisms of tumor multidrug resistance are complex, and therefore, the actual clinical significance of these mechanisms is unknown and has not yet been more substantiated. The best means to study tumor drug resistance is to obtain human tumor tissues for research. Many challenging factors limit the development of natural anticancer biomolecules as drug products. First, the development of natural products is a complex and time-consuming process, such as extraction, purification, isolation, characterization, etc. Moreover, in addition to toxic side effects, reduced water solubility, decreased absorption, and lack of selectivity for targeting cancer cells are the main obstacles to the development of anticancer drugs with natural origins. In recent years, China’s high-throughput technology has made great progress in the field of biomedicine, using high-precision technology, combined with modern pharmacology, pharmacodynamics, molecular biology, and other new technologies, to study the molecular mechanisms of tumors. It is expected that in the future, standardized treatment protocols in oncology will allow for the use of multiple compounds of natural origin to improve clinical efficacy and reduce the side effects of anticancer therapies. Two methods of prevention have been identified in the fight against cancer. One is chemoprevention, and the other is immunoprevention [300]. Currently, the best method is considered to be immunoprophylaxis. However, its potential side effects, toxicity, mutations, and immune checkpoint modulations remain significant issues and limitations that prevent this method from reaching clinical application [301]. We believe that with the further systematization and comprehensiveness of research on immunity or the molecular pathology of cancer, natural products of plant origin will be widely applied in clinical medicine. 

## Figures and Tables

**Table 1:** Effect and mechanism of MDR gene-related proteins of plant-derived natural products on drug-resistant cancer.

Chemical Family	Molecule Name	Concentration	Resistant Cancer	Cell Line and In Vivo Model	Mechanism	Reference
Flavonoids	Quercetin	33 μmol/L	Doxorubicin resistant human colon cancer	SW620/Ad300	Increased: accumulation of drug, apoptosis	[137]
	Rutin	20 μmol/L	Human breast cancer	MDA-MB-231 MCF-7	Decreased: P-gP	[138]
	Myricetin	25 μmol/L	Ovarian cancer	A2780 OVCAR3	Increased: Bax, cleaved caspase 3 Decreased: Bcl-2, MDR1	[139]
	Naringin	129.77 μmol/L	Cisplatin resistant human lung cancer	A549/DDP	Increased: Bax, cleaved caspase 3 Decreased: Bcl-2, P-gP, MRP1, *p*-AKT, CXCR4	[140]
		10–40 μmol/L	Cisplatin resistant ovarian cancer	SKOV3/CDDP	Decreased: NF-κB, P-gP	[141]
	Apigenin	5–80 mol/L	Cisplatin resistant human lung cancer	A549/DDP	Decreased: LRP, P-gP	[142]
	Silibinin	60 μmol/L	Multidrug resistant small-cell lung carcinoma	VPA17	Decreased: drug efflux	[143]
Polyphenols	Hyperin	12.5 μmol/L	Vincristine resistant colorectal cancer	HCT8/VCR	Decreased: P-gP	[144]
	(-)-Epigallocatechin 3-gallate	15.81 µg/ml	Cisplatin resistant cervical cancer	SiHa/DDP	Decreased: P-gP, ABCG2	[145]
	Curcumin	2.5 µmol/L	Doxorubicin resistant human leukemia	K562/DOX	Increased: intracellular DOX Decreased: S100A8, P-gP	[146]
	Naringenin	100 μmol/L	Doxorubicin resistant human breast cancer	MCF-7/DOX	Increased: uptake of 5-CFDA, doxorubicin	[147]
	Icaritin	0–30 μmol/L	Adriamycin resistant human hepatoma	HepG2/ADR	Decreased: MDR gene, P-gP	[148]
Quinones	Emodin	0–20 μmol/L	Adriamycin resistant human myelogenous myeloid leukemia	K562/ADM	Increased: intracellular accumulation of rhodamine 123 Decreased: P-gP	[149]
		40 mg/kg	Gemcitabine resistant human pancreatic cancer mice model	gemcitabine-resistant PANC-1 cell	Decreased: MDR1, P-gP, MRP1, MRP5	[150]
		50 µmol/L	Platinum resistant ovarian cancer	COC1/DDP	Decreased: P-gP	[151]
	artesunate	25, 50 mg/kg	drug-resistant esophageal cancer	Eca109/ABCG2, Xenografts of Eca109/ABCG2 in BALB/c nu/nu mice	Increased: apoptosis Decreased: Tumor volume, ABCG2	[157]
Terpenoids	β-elemene	20–320 µmol/L	Cisplatin resistant ovarian cancer	SKOV3/DDP	Decreased: ABCB1, LRP, P-gP	[152]
		31.92 µg/mL	Daunorubicin resistant on human leukemia	K562/DNR	Decreased: P-gP	[153]
		22.02 µg/mL	Doxorubicin resistant on human leukemia	K562/DOX	Decreased: P-gP	[154]
Alkaloids	Sinomenine	100, 200 µg/mL	Doxorubicin resistant human bladder cancer	253J/DOX	Increased: cleaved PARP, Bax Decreased: P-gP	[155]
	Matrine	32.5 µg/mL	Adriamycin resistant human bladder cancer	BIU-87/ADM	Decreased: P-gP, adriamycin efflux	[156]
	Berberine	BGC-823/DDP: 549.6 µmol/L SGC-7901/DDP: 562.1 µmol/L	Cisplatin resistant gastric cancer	BGC-823/DDP, SGC-7901/DDP, Xenografts of SGC-7901/DDP in BALB/c-nu mice	Increased: c-caspase 3, c-caspase9, Bax Decreased: MDR1, MRP1, and PI3K/AKT/mTOR signaling pathway related protein	[158]
		20 μg/mL	doxorubicin resistant breast cancer	MCF-7/DoxFluc, Xenografts of MCF-7/DoxFluc in BALB/c nude mice	Decreased: P-gP/ABCB1, MRP1/ABCC1	[159]
Compounds	Zuojinwan	1027.5–4110 mg/kg	drug-resistant cancer	HCT116/L-OHP, SGC7901/DDP, Bel/Fu, Xenografts of HCT116/L-OHP in male athymic nude mice	Decreased: P-gP	[160]

Abbreviation: caspase 3; cysteine-aspartic-acid-specific protease-3, MDR; multiple drug resistance, MRP1: multidrug resistance-associated protein 1, CXCR4; CXC chemokine receptor-4, NF-κB; nuclear factor kappa-lightchain-enhancer of activated B cells, ABCG2; ATP-binding cassette subfamily G memberII, ABCB1; ATP binding cassette subfamily B member 1, PARP; polyadenosine diphosphate ribose polymerase 1.

**Table 2:** Effect and mechanism of Apoptosis related pathway of plant-derived natural products on drug-resistant cancer.

Chemical Family	Molecule Name	Concentration	Resistant Cancer	Cell Line and In Vivo Model	Mechanism	Reference
Polyphenols	Grape seed procyanidin	10–40 µmol/L	Paclitaxel-resistant ovarian cancer	A2780/T	Increased: accumulation of Rho-123, p65, YB-1 Decreased: P-gP, phospho-AKT, phospho-IκBα, *p*-ERK1/2	[49]
	Baicalin	5, 10 μmol/L	Adriamycin-resistant leukemia cell	HL-60/ADM	Increased: cleaved PARP, cleaved caspase 3 Decreased: MRP1, LRP, Bcl2, *p*-AKT	[163]
	Wogonin	20–80 μmol/L	Human colon cancer under hypoxic conditions	HCT116	Decreased: HIF-1α, HKII, PDHK1, LDHA, *p*-PI3K, *p*-AKT	[164]
	Tanshinone IIA	20 µg/mL	Doxorubicin-resistant human breast cancer	MCF-7/DOX	Decreased: P-gP, BCRP, MRP1	[165]
	Resveratrol	45.9 µmol/L	Adriamycin-resistant human leukemia	K562/ADR	Increased: caspase 3 and caspase 8 Decreased: P-gP, MDR1, *p*-AKT	[166]
	Curcumin	0–45 µmol/L	Adriamycin-resistant mouse leukemia cell	L210/ADR	Decreased: P-gP, *p*-AKT at Ser-473, *p*-GSK-3b at Ser-9	[167]
	Nobiletin	31.62 µmol/L	Paclitaxel-resistant ovarian cancer	A2780/T	Increased: P53 Decreased: AKT, *p*-AKT, *p*-ERK1/2, Nrf2	[168]
		50 µmol/L	Adriamycin-resistant human non-small-cell lung cancer	A549/ADR	Decreased: MRP1, MYCN, β-catenin, *p*-GSK-3β, Bcl-2, survivin	[169]
Quinones	Emodin	9 μmol/L	5-fuorouracil-resistant human colorectal carcinoma	SW480/5-Fu	Increased: cleaved caspase 3, Bax Decreased: Bcl-2, *p*-ERK, *p*-AKT	[170]
	Levoshikonin	3.92 μmol/L	Cisplatin-resistant cervical cancer	Hela/DDP	Increased: Bax, Cleaved caspase 3 Decreased: Bcl-2	[171]
	1,4-Naphthoquinone	0.1 µmol/L	Vincristine-resistant chronic myeloid leukemia Daunorubicin-resistant chronic myeloid leukemia	K-562-Lucena1 FEPS	Increased: H2AFX, ABCB1	[172]
Terpenoids	Triptolide	10–160 nmol/L	Cisplatin-resistant cervical cancer	Hela/DDP	Decreased: Bcl-2, XIAP	[173]
	Cucurbitacin B	15 nmol/L	Gefitinib-resistant lung cancer	A549-GR	Increased: E-cadherin, ROS Decreased: EGFR, *N*-cadherin, Vimentin, *p*-PI3K, *p*-AKT, *p*-mTOR	[174]
	β-elemene	20, 40 µg/mL	Cisplatin-resistant human lung cancer	A549/DDP	Decreased: MDR1, LRP, *p*-PI3K, *p*-AKT	[175]
		5–100 µg/mL	Adriamycin-resistant gastric cancer	SGC7901/Adr, Xenografts of SGC7901/Adr in BALB/c nude mice	Increased: caspase 3	[182]
Flavonoids	Quercetin	0.005–0.15 µg/mL	Cisplatin-resistant cervical cancer	SiHa/DDP	Decreased: AKT, mTOR, p70S6K, P-gP	[176]
	Luteolin	10–200 mg/L	Doxorubicin-resistant cervical cancer	HeLa/DOX	Increased: PI3K, Cleaved caspase 3 Decreased: *p*-AKT, *p*-mTOR, p70S6K, Ki67	[177]
Alkaloids	Matrine	0.15–1.2 mg/mL	Adriamycin-resistant human breast cancer	MCF-7/ADR	Increased: PTEN, Bax, cleaved caspase 3 Decrease: MDR1, MRP1, *p*-AKT, Bcl-2	[178]
Furanocoumarin	Imperatorin	2 µmol/L	Cisplatin-resistant cervical cancer	Hela/R	Increased: Bim, Bak, Bax, release of cytochrome c, activation of caspase 3	[179]
Compound	Metformin	0.25–8 mmol/L	Cisplatin-resistant endometrial cancer	Ishikawa/DDP	Increased: Bax Decreased: Bcl-2, AKT1	[180]
		10–80 mmol/L	Progestin-resistant endometrial cancer	MPA-R-Ishikawa	Decreased: proliferation	[181]
	Sijunzi Tang	4.806 mg/mL	Gefitinib-resistant non-small cell lung cancer	PG9-GR, Xenografts of PC9/GR in BALB/c nu/nu mice	Increased: Bax, Decreased: Bcl-2	[183]
	Compound Zhebei	2.5–10 g/kg	Oxaliplatin-resistant colon cancer	Xenografts of HCT-116/L-OHP in BALB/c nude mice	Increased: Bax, Decreased: Bcl-2	[184]
	Compound Zhebei	2.5–10 g/kg	Cisplatin-resistant acute lymphoblastic leukemia	Xenografts of L1210 in DBA/2 nude mice	Increased: Bax, Decreased: Bcl-2	[185]

Abbreviation: YB-1; Y-box-binding protein-1, ERK; extracellular signal-regulated kinases, HIF-1; hypoxia-inducible factor 1, HKII; hexokinase II, PDHK1; pyruvate dehydrogenase kinase, LDHA; lactate dehydrogenase A, GSK; glycogen synthase kinase, Nrf2; nuclear factor E2-related factor 2, H2AFX; H2A histone family, member X, XIAP; X-linked inhibitor of poptosis protein, mTOR; mammalian target of rapamycin.

**Table 3:** Effect and mechanism of Autophagy related pathway of plant-derived natural products on drug-resistant cancer.

Chemical Family	Molecule Name	Concentration	Resistant Cancer	Cell Line and In Vivo Model	Mechanism	Reference
Flavonoids	Isoliquiritigenin	0–100 µmol/L	Doxorubicin resistant human uterine sarcoma	MES-SA/Dx5 MES-SA/Dx5-R	Increased: cleaved PARP, cleaved caspase 7, LC3BI, LC3BII Decreased: PARP, Bcl-2, caspase 7, *p*-mTOR, SQSTM1/p62	[188]
	Baicalein	0–10 µg/mL	5-Fluorouracil resistant hepatocellular carcinoma	Bel7402/5-Fu	Decreased: P-gP, Bcl-xL	[189]
	Baicalin	20–40 µmol/L	Cisplatin resistant cervical cancer	*C*-33A/Cis	Increased: ratio of MAPLC3II/MAPLC3I, Beclin-1, Atg5, Atg12	[191]
	Puerarin	25 µmol/L	Adriamycin resistant human leukemia	K562/ADR	Increased: LC3-II, Beclin-1, caspase 3, accumulation of adriamycin Decreased: MDR1, NF-κB activity, cyclingB1, *p*-AKT, *p*-JNK	[193]
	Hesperetin	50–200 µmol/L	Cisplatin resistant ovarian adenocarcinoma	A2780/DDP	Increased: p62, p53, Bax, Caspase 3 Decreased: LC3, Beclin1, AMPK, mTOR, Bcl-2	[194]
Alkaloids	Matrine	0.2 mg/mL	Adriamycin resistant human leukemia	K562/ADM	Increased: LC3II Decreased: p62	[195]
	Lycorine	6 µmol/L	Imatinib resistant human leukemia	K562/IM	Increased: p62 Decreased: Beclin-1, Atg5, LC3-II, P-gP	[196]
	Cepharanthine	8 µmol/L	Icotinib resistant non-small cell lung cancer	PC-9/IR	Increased: p53, Beclin-1, ratio of LC3-II/LC3-I Decreased: ratio of *p*-mTOR/mTOR	[197]
	Berberine	100 µmol/L	Doxorubicin resistant breast cancer	MCF-7/ADR, Xenografts of MCF-7/ADR in BALB/c nude mice	Increased: p62, Akt, mTOR; Decreased: MDR1, LC3-II/I, PTEN	[203]
Terpenoids	β-elemene	15 µg/mL	Cisplatin resistant human lung cancer	SPC-A-1/DDP	Increased: Beclin-1 Decreased: MRP-1, P-gP	[198]
		120 µg/mL	Gefitinib resistant non-small cell lung cancer	PC9GR HCC827GR	Increased: LC3B-II, SQSTM1 Decreased: METTL3	[199]
	Triptolide	0–100 nmol/L	Cisplatin resistant ovarian cancer	SKOV3/DDP	Increased: E-cadherin, Beclin1, LC3, ratio of LC3II/LC3-I Decreased: *N*-cadherin, MMP9	[200]
Polyphenols	Naringenin	15–240 mg/L	Cisplatin resistant cervical cancer	Hela/DDP	Increased: ratio of *p*-AMPK/AMPK, Bax, Beclin1, LC3II Decreased: LC3I	[201]
Compound	Extract of Scutellaria baicalensis	0–400 µg/mL	Cisplatin resistant Ovarian cancer	A2780Cis	Increased: Atg12 Decreased: p53, DRAM, Beclin 1, Atg5	[190]
	Kang’ai injection	100.07 g/L	Cisplatin resistant Human Lung Cancer	A549/DDP	Increased: Atg7, LC3I, LC3II, Beclin 1, ratio of Bax/Bcl-2, cleaved caspase 3	[202]

Abbreviation: LC3; light chain 3, METTL; methyltransferase-like protein, SQSTM; sequestosome 1, AMPK; adenosine monophosphate-activated protein kinase, DRAM; DNA damage-regulated autophagy modulator 1.

**Table 4:** Effect and mechanism of MAPK signaling pathway of plant-derived natural products on drug-resistant cancer.

Chemical Family	Molecule Name	Concentration	Resistant Cancer	Cell Line and In Vivo Model	Mechanism	Reference
Polyphenols	Hesperetin	0.6–160 µmol/L	Cisplatin resistant human lung cancer	A549/DDP	Decreased: P-gP, ratio of *p*-P65/t-P65, ratio of P-IκB/IκB	[205]
	Resveratrol	100 µmol/L	Adriamycin resistant human Osteosarcoma	U2OS/ADR	Decreased: P-gP, acetylated p65, *p*-p38	[216]
	Rosmarinic acid	46.47 µg/mL	Cisplatin resistant non-small cell lung cancer	A549/DDP	Increased: cleaved caspase 3, Bax, p53, p21, *p*-C-Jun Decreased: Bcl-2, caspase 3, P-gP	[217]
	Baicalin	8 µg/mL	Cisplatin resistant human lung cancer	A549/DDP	Decreased: MARK2, *p*-AKT	[218]
	Curcumin	20 μmol/L	Vincristine resistant esophageal carcinoma	Eca-109/VCR	Decreased: p38MAPK, *p*-p38MAPK, ERCC1, P-gp	[219]
		0–60 µg/mL	Adriamycin resistant hepatocarcinoma	HepG2/ADM	Decreased: p38MAPK	[220]
	Paeonol	BGC-823/AP: 53.64 mg/L MGC-803/AP: 56.83 mg/L	Apatinib resistant gastric cancer	BGC-823/AP MGC-803/AP	Decreased: HK2, GLUT1, LDHB, MAPK1	[221]
Lignans	Schisandrin A	20 µmol/L	Doxorubicin resistant human breast cancer	MCF-7/DOX	Increased: Cleaved caspase 9 PARP Decreased: P-gP	[209]
Lignans	Muscone	0–16 µmol/L	cisplatin resistant lung cancer	A549/DDP, Xenografts of A549/DDP in BALB/c-nu mice	Decreased: MRP1, P-gP, p50, p52, p65	[225]
Alkaloids	Matrine	0–300 µmol/L	Adriamycin resistant human leukemia	K562/ADR	Increased: cleaved caspase 9 Decreased: ABCB1, ABCC1, ABCG2, phosphorylation of NF-κB, survivin, Bcl-xL	[206]
	Tetrandrine	0–8 μmol/L	Vincristine resistant gastric cancer	SGC-7901/VCR	Increased: cleaved-caspase 9, cleaved-caspase 3, *p*-p38, *p*-JNK, activation of PARP Decreased: Bcl2, *p*-ERK, *p*-CDC2	[224]
Terpenoids	Furanodiene	0.200 µmol/L	Doxorubicin resistant breast cancer	MCF-7/DOX	Increased: Bad, caspase 3/7/8, PARP, p65, IKKα/β Decreased: Bcl-xl	[207]
	Triptolide	0.03–3 µmol/L	Taxol resistant human lung adenocarcinoma	A549/Taxol	Decreased: NF-κB, P62, FLIP, XIAP, Bcl-2, Bcl-xL, COX-2	[208]
	Rotundioic acid	0–4 µg/mL	Adriamycin resistant human leukemia	K562/ADM	Increased: *p*-p38,p-ERK1/2 Decreased: MDR1, P-gP	[222]
	Isolinderalactone	HCT116-OxR: 5.13 μmol/L HT29-OxR: 9.46 μmol/L	Oxaliplatin resistant colorectal cancer	HCT116-OxR HT29-OxR	Increased: p21, p27, GRP78, CHOP, DR4, DR5, *p*-JNK, *p*-p38, Bim, Bax Decreased: cyclinB1, CDC2, Bid, Bcl-2, Bcl-xL	[223]

Abbreviation: JNK; c-Jun *N*-terminal kinase, CDC2; cell division cycle 2, GRP78; glucose-regulated protein 78, CHOP; C/EBP homologous protein, DR4, DR5; death receptor 4/5, Bim; B-cell lymphoma 2-interacting mediator of cell death, Bid; BH3-interaction domain death agonist, GLUT1; glucose transporter 1, LDHB; lactate dehydrogenase B, MAPK; mitogen-activated protein kinase.

**Table 5:** Effects and mechanisms of plant-derived natural products on drug-resistant cancer.

Chemical Family	Molecule Name	Concentration	Resistant Cancer	Cell Line	Mechanism	Reference
Quinones	Shikonin	4 μmol/L	Cisplatin-resistant ovarian cancer	SKOV3/DDP	Increased: P18, Bax, cleaved caspase 3 Decreased: cyclin D1, CDK2, ratio of *p*-Rb/Rb, Bcl-2	[230]
	Plumbagin	90.8 μmol/L	Cisplatin-resistant human cervix squamous carcinoma	A431/Pt	Increased: ROS, *p*-Rb, sub G0 phase	[231]
Alkaloids	Tetramethypyrazine	73 µmol/L	Multidrug-resistant human bladder cancer	Pumc-91/ADM T24/DDP	Increased: Topo-II, S phase Decreased: MRP1, GST, BCL-2	[232]
	Homoharringtonine	18.57 ng/mL	Vemurafenib-resistant melanoma	A375R	Increased: G0/G1 phase Decreased: Cyclin E1, CDK2, IRS4, PI3K, *p*-Akt, *p*-ERK	[233]
Terpenoids	Triptolide succinate monoester	33.32 µmol/L	Cisplatin-resistant human lung adenocarcinoma	A549/DDP	Increased: G2/M, apoptosis	[234]
	Kamebakaurin	62.97 μmol/L	Adriamycin-resistant hepatocellular carcinoma	HepG2/ADM	Increased: Bax, *p*-PTEN, G2 phase Decreased: Bcl-2, MDR1, *p*-AKT	[235]
Saponins	Ginsenoside Rh2	20 µmol/L	5-fuorouracil-resistant human colorectal carcinoma	HCT-8/5-Fu LoVo/5-Fu	Increased: cleaved-caspase 3, *p*-IκB, E-cadherin, G1 phase Decreased: cyclin D1, CDK2, *p*-Rb, Bcl-2, *N*-cadherin, Vimentin, MMP9, MRP1, MDR1, LRP, GST	[236]
Polyphenols	Curcumin	13.69 μmol/L	NNMT-related resistant colorectal cancer	SW480/NNMT	Increased: *p*-Rb, G2/M Phase, ROS Decreased: *p*-STAT3, CDK1, CDK2	[237]
Flavonoids	Isoxanthohumol	42.1 μmol/L	Cisplatin-resistant human lung adenocarcinoma	A549/DDP	Increased: G0/G1 phase Decreased: P-gP, LRP, MRP, PI3K, *p*-AKt	[238]
Compound	Metformin	28. 02 μg/ml	Oxaliplatin-resistant human gastric cancer	SGC-7901/L-OHP	Decreased: cyclin D1	[239]
	Buzhong yiqi decoction	0.25 g/mL	Cisplatin-resistant human gastric cancer	SGC-7901/CDDP	Increased: G1/S phase Decreased: survivin, Bcl-2, NF-κB	[240]
	Digitoxin	132.65 nmol/L	Adriamycin-resistant human liver cancer	HepG2/ADM	Increased: cyclin B1, numbers of punctuate γH2AX foci, *p*-CHK2, *p*-ATR(Ser428), Bax, G2/M phase Decreased: *p*-CDK1 (Thr14), CDC25C, Bcl-2	[241]

Abbreviation: CDK; cyclin-dependent kinase, Rb; retinoblastoma, ROS; reactive oxygen species, GST; glutathione S-transferase, IRS4; insulin receptor substrate 4, STAT3; signal transducer and activator of transcription 3, CHK2; cell cycle checkpoint kinase 2, CDC25C; cell division cycle 25 homologus protein C.

**Table 6:** Effects and mechanism of plant-derived natural products on resistant cancer.

Chemical Family	Molecule Name	Concentration	Resistant Cancer	Cell Line	Mechanism	Reference
Flavonoids	Luteolin	20 μmol/L	Paclitaxel-resistant esophageal cancer	TE-1/PTX EC109/PTX	Increased: E-cadherin Decreased: Slug, *N*-cadherin, Vimentin	[243]
		0–40 μmol/L	Paclitaxel-resistant esophageal cancer	TE-1/PTX EC109/PTX	Increased: *p*-PI3K, *p*-AKT, E-cadherin Decreased: SOX2, OCT4, NANOG	[244]
	Quercetin	0.7 µmol/L	Doxorubicin-resistant human breast cancer	MCF-7/dox	Increased: PTEN Decreased: CD44^+^/CD24^−/low^, *p*-AKT	[245]
	Genistein	10 μmol/L	Multidrug-resistant liver cancer	SMMC-7721	Decreased: CD133, CD44, Gli1, ABCG2	[254]
Alkaloids	Matrine	10 mg/mL	5-Fuorouracil-resistant colon cancer stem cells	HCT116/5-Fu	Increased: apoptosis Decreased: ABCB1, *p*-PI3K, *p*-Akt, *p*-mTOR, P-gP	[246]
	Neferine/isoliensinine	Neferine: 6.5 μmol/L Isoliensinine: 12.5 μmol/L	Cisplatin-resistant colon cancer stem cells	HCT-15/DDP-CSCs	Increased: ROS, Bax, cytochrome c, caspase 3, PARP, sub G0 phase Decreased: PI3K, *p*-AKT, mTOR, BCl-2	[247]
Terpenoids	Ursolic acid	9–32 μg/g	Drug resistance in nude mice bearing ovarian cancer stem cells	Skov3-sp	Decreased: ABCG2	[248]
	Norcantharidin	A2780^Cis^: 1.46 μg/mL SKOV3^Pac^: 1.8 μg/ml	Drug-resistant ovarian cancer	A2780^Cis^ SKOV3^Pac^	Increased: Ptch1 Decreased: Sox2, Nanog, Bmi1, Shh	[255]
Polyphenols	Curcumin	10, 20 μmol/L	Drug-resistant colon cancer	HCT116/OX HCT8/5-Fu	Decreased: CD133, ALDH1, CD24	[249]
		10 μmol/L	5-Fuorouracil-resistant gastric cancer	BGC-823/5-Fu	Increased: E-cadherin, GSK-3β Decreased: Wnt2, β-catenin, vimentin	[257]
Compound	Yi-qi-hua-yu-jie-du decoction	2.5 g/mL	5-Fuorouracil-resistant gastric cancer	BGC823/5-Fu	Decreased: P-gP, MRP1, Nrf2, *p*-PI3K, *p*-AKT	[250]

Abbreviations: SOX2; sex-determining region Y-box2, OCT4; octamer-binding transcription factor, Ptch1; patched 1, Bmi1; B cell-specific Moloney murine leukemia virus insertion site 1, Shh; sonic hedgehog, GSK-3β; glycogen synthase kinase-3β.

**Table 7:** Effect and mechanism of Other Signaling pathways of plant-derived natural products on resistant cancer.

Chemical Family	Molecule Name	Concentration	Resistant Cancer	Cell Line and In Vivo Model	Mechanism	Reference
Polyphenols	Baicalin	0–100 µmol/L	Human erythroleukemia	K562	Increased: cleaved Notch1	[258]
Alkaloids	Sinomenine	40 µmol/L	Cisplatin-resistant cervical cancer	Hela/DDP	Decreased: Notch1, STAT3, Hes1	[259]
	Matrine	1–32 μmol/L	Cisplatin-resistant lung cancer	A549/DDP	Increased: E-cadherin Decreased: Vimentin, Slug, *p*-p65	[265]
	Berberine	25 µmol/L	EGFR-TKI-resistant lung cancer	A549,H1975	Increased: E-cadherin, Decreased: vimentin, Snail	[272]
Flavonoids	Quercetin	128 μmol/L	Gemcitabine-resistant non-small-cell lung cancer	A549/Gb	Increased: E-cadherin, miR-101 Decreased: *N*-cadherin, Vimentin, EZH2, TGF-β1, ratio of *p*-SMAD4/SMAD4	[263]
Polysaccharide	Astragalus polysaccharides	0.3 g/kg/d	Cisplatin-resistant lung adenocarcinoma BALB/c nude mice	A549/DDP	Increased: α-catenin Decreased: quality of tumor, *N*-cadherin, LRP, MRP, P-gP	[264]
Terpenoids	Celastrol	0–4 μmol/L	Cisplatin-resistant nasopharyngeal carcinoma	Cis-039 Cis-BM	Increased: cleaved caspase 3, cleaved caspase 8, cleaved caspase 9, PARP, Bax, Bim S, *p*-p38, *p*-ERK1/2, *p*-JNK1/2, Decreased: Bcl-xL, Bcl-2	[266]
	Oridonin	5 μmol/L	Fulvestran-resistant breast cancer	MCF-7/Ful	Increased: γ-H2AX, Bax, caspase 3, caspase 9, ratio of *p*-CDC2/CDC2, ratio of LC3-II/I, G0/G1 phase Decreased: Bcl-2, cyclin D1	[267]
Terpenoids	β-Elemene	20 μg/mL	Cisplatin-resistant human lung adenocarcinoma	A549/DDP	Increased: apoptosis, ROS, released of GSH, accumulation of intracellular Rh-123 Decreased: the mitochondrial membrane potential (ΔΨm), P-gP	[268]
		108.5 μg/mL	Paclitaxel-resistant lung cancer	A549/Taxol	Increased: Bax, caspase 3 Decreased: JAK2, STAT3, *p*-STAT3, Bcl-2	[269]
		1.5 µg/mL	Adriamycin-resistant gastric cancer	SGC7901/ADR, Xenografts of SGC7901/Adr in BALB/c-nu/nu mice	Increased: E-cadherin. Cb1-b, Decreased: Vimentin, MMP2, MMP9, ZEB1, ZEB2, EGFR, *p*-EGFR, *p*-AKT, *p*-ERK	[273]
Quinones	Shikonin	9 µmol/L	Cisplatin-resistant human ovarian cancer	A2780-CR	Increased: Bax, *p*-ERK1/2, ERK2, *p*-JNK1/2, JNK1/2, *p*-p38, p38, E-cadherin Decreased: Bcl-2, *N*-cadherin	[270]
		0.4 µmol/L	Cisplatin-resistant bladder cancer	T24R	Increased: *p*-RIP3, PUMA, Bax Decreased: Bcl-2	[271]
Compound	Buzhong Yiqi Decoction	1.46–5.82 g/kg	Cisplatin-resistant lung cancer	A549/DDP, Xenografts of A549/DDP in BALB/c nu/nu mice	Decreased: β-catenin	[274]
	Jiedu Fuzheng prescription	52, 104 g/kg	Gefitinib-resistant non-small cell lung cancer	Xenografts of PC9/GR in BALB/c nu/nu mice	Decreased: *p*-ERK/ERK, Nrf2	[275]

Abbreviations: Notch; Notch homolog, Hes1; Hairy and Enhancer of Split 1, Slug; SNAI2, EZH2; Enhancer of Zeste Homolog 2, TGF-β; Transforming Growth Factor-β, SMAD4; SMAD Family Member 4, H2AX; H2A Histone Family Member X, GSH; Glutathione, RIP3; Receptor Interacting Protein Kinase 3, ZEB1: zinc finger E-box-binding protein1, ZEB2: zinc finger E-box-binding protein 2, PUMA; p53 Upregulated Modulator of Apoptosis.

## Data Availability

Not applicable.
